# Polyethylene-poly(methyl acrylate) Block Copolymers from PACE-SARA ATRP: Utilizing Polyolefin Active Ester Exchange-Based Macroinitiators in Atom Transfer Radical Polymerization

**DOI:** 10.1021/acs.macromol.4c02684

**Published:** 2025-01-30

**Authors:** Khidong Kim, Jacobo Strong, Stephen Don Sarkar, Dung Nguyen, Huong Dau, D.A. Anwar Al-Aman, Sajjad Dadashi-Silab, Eva Harth, Krzysztof Matyjaszewski

**Affiliations:** † Department of Chemistry, Carnegie Mellon University, Pittsburgh, Pennsylvania 15213, United States; ‡ Department of Chemistry, Center of Excellence in Polymer Chemistry (CEPC), University of Houston, Houston, Texas 77204, United States

## Abstract

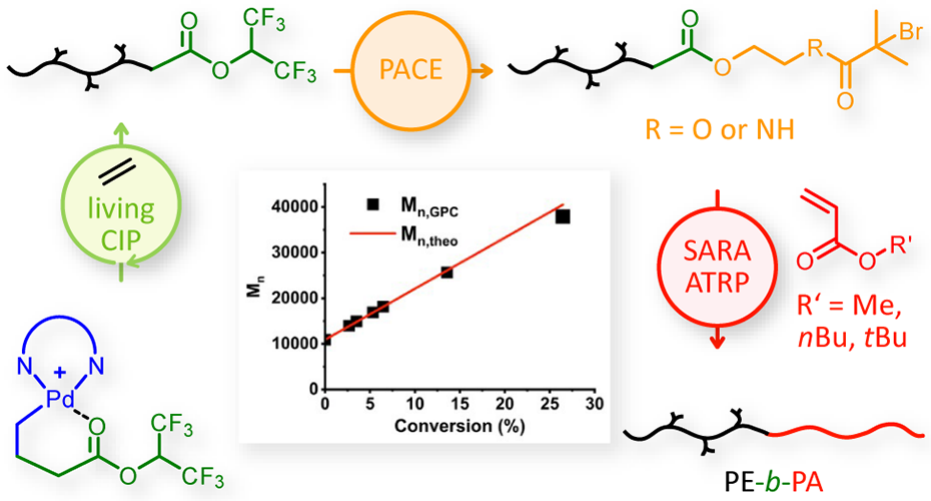

Accessing a facile pathway to prepare polyolefin-polar block copolymers with low dispersity and high control remains a challenge due to the distinct polymerization pathways of the composing blocks. This study utilized the polyolefin active ester exchange, the PACE approach, as a viable solution. The PACE approach, using palladium-catalyst-based coordination-insertion polymerization, was combined with SARA ATRP (supplemental activator/reducing agent atom transfer radical polymerization). A single-chain-end active ester functionalized polyethylene (PE) was produced from an α-diimine Pd(II) hexafluoroisopropyl ester chelate complex, which facilitated a living polymerization of ethylene. Transesterification with 2-hydroxyethyl α-bromoisobutyrate (HOBIB) or 2-hydroxyethyl α-bromoisobutyramide (HOBIBA) formed α-bromoisobutyrate or α-bromoisobutyramide chain-end-functionalized polyethylene. The approach resulted in controlled synthesis of polymers with low dispersity (*Đ*), high initiation efficiency, and high reproducibility. Both the amide-linked and ester-linked macroinitiators showed >90% initiation efficiency and *Đ* values of block copolymers as low as 1.05. This work demonstrated a successful combination of two living polymerization techniques, an insertion and controlled radical polymerization, unified in PACE-SARA ATRP, offering access to polyolefin-containing block copolymers with chemically distinct structures.

## 1 Introduction

Recent studies of polymer architecture provided insights into the characteristics of different polymer structures.^1^ Of particular interest are block copolymers, BCP, which can efficiently self-assemble.^2,3^ BCP synthesis dates to the 50s which involves connecting distinct polymeric segments through covalent bonds.^4^ Initially, a single mechanism was employed to synthesize the comprising blocks^5−9^ such as sequential carbocationic block copolymerization,^10^ anionic polymerization,^4,9^ atom transfer radical polymerization (ATRP),^5,11−13^ and ring-opening metathesis polymerization (ROMP).^14,15^

Previously, two different synthetic approaches were also sequentially employed to allow access to block copolymers based on different monomers.^16^ Some examples include switching from anionic to cationic polymerization,^17−19^ cationic to anionic polymerization,^20−22^ cationic polymerization to ATRP,^23−25^ ATRP to cationic polymerization,^26−28^ anionic polymerization to ATRP,^29−31^ and ROMP to ATRP.^32,33^ Two separate polymerization processes may also occur simultaneously.^34^

However, the combination of olefins and acrylates in block copolymer structures has been more challenging due to the different reactivities of the monomers and propagating species. Polyolefins are typically prepared via coordination–insertion polymerization,^35−37^ whereas polar vinyl polymers are formed by reversible-deactivation radical polymerization (RDRP).^38^ Approaches such as ATRP^5,38,39^ are widely used to synthesize well-defined polar homopolymers.^5,12,40−42^

Although late transition metal complexes are more tolerant toward polar monomers, only a small fraction of polar monomers can be incorporated during copolymerization.^43^ In fact, the coordination-insertion process of olefins is retarded in the presence of polar monomers.^44,45^ Conversely, the polymerization of olefinic monomers is limited to a few units with the ATRP procedures; the dormant chain ends in polyolefins cannot be efficiently activated in controlled radical polymerization.^46,47^ Hence, synthesizing polyolefin-polar BCPs has been challenging due to the differences in the monomer and chain end reactivities.^48,49^

Despite these challenges, BCPs with polyolefin and polar vinyl blocks are appealing.^50−52^ With polyolefin-polar BCPs, the advantageous chemical, physical, and thermal properties of polyolefins can be combined with a wide array of functionalities associated with the polar vinyl blocks.^48,53^ Prior attempts had limitations in integrating the olefinic and polar blocks; the chain-walking process permitted the selective incorporation of a single monomer into the polyolefin chain end. However, the continued polymerization of the further acrylic monomers was effectively halted.^43,54^

Previous efforts were concerted to expanding the range of compatible monomers for the coordination–insertion polymerization.^55^ When using a single pathway for different blocks, harsh conditions^56^ and side reactions^57^ were often involved. Thus, the paradigm shifted from the “1-for-2 strategy”^58^ (one approach for two blocks) to the “2-for-2 strategy” (two approaches for two blocks).^49,59−61^ The latter strategy involved the “switching” of the polymerization approach from the coordination–insertion polymerization to the reversible-deactivation radical polymerization. This strategy fused two distinct synthesis approaches. For example, a recent study^62^ suggested the possibility of preparing polyolefin chains terminated with phenyl groups. The functionalization of polyolefin chains with phenyl groups can yield hetero- and homotelechelic poly(α-olefins). The phenyl group can undergo postpolymerization reactions and serve as the initiator to prepare di- and triblock copolymers.^62^

**Figure 1:**
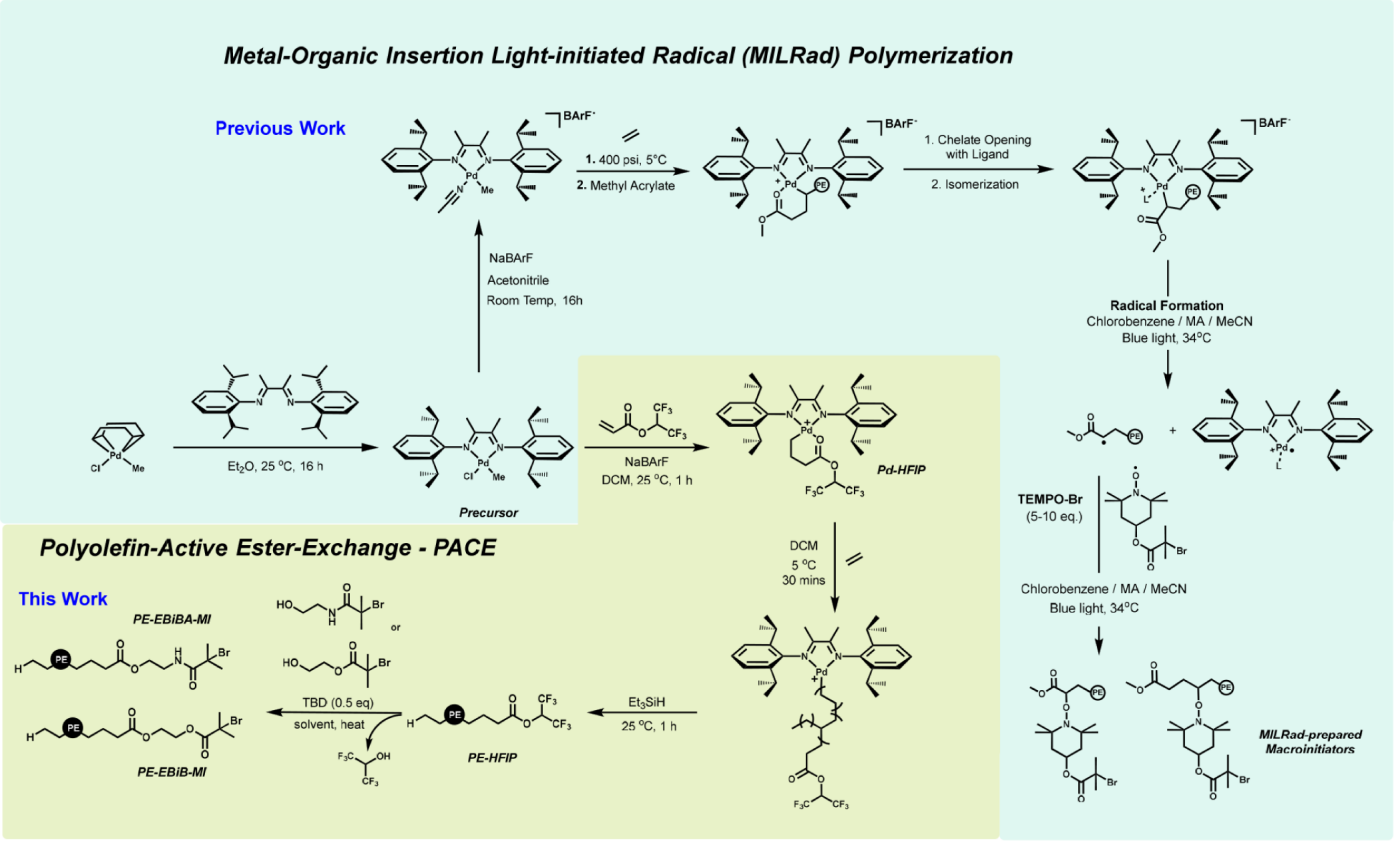
A general synthetic scheme for preparing macroinitiators via MILRad functionalization and the PACE process. MILRad functionalization (blue). Homolytically cleaved polyethylene-radical species were captured by TEMPO-Br. PACE (yellow) Following ethylene polymerization with hexafluoroisopropyl ester terminated Pd-centered macrochelate (Pd-HFIP), transesterification with 2-hydroxyethyl α-bromoisobutyrate (HOBIB) or 2-hydroxyethyl α-bromoisobutyramide (HOBIBA) yielded the PACE-SARA ATRP macroinitiators PE-EBiB-MI and PE-EBiBA-MI. HFIP: hexafluoroisopropyl.

Recently, the MILRad (Metal–Organic Insertion Light-Initiated Radical Polymerization) approach was introduced.^60,63−65^ Under the MILRad approach, the polyolefin blocks were synthesized via coordination–insertion polymerization, followed by the addition of a polar monomer to form a macrochelate. As shown in Figure 1, under the MILRad polymerization strategy, light irradiation formed the macroradical that could (1) undergo free radical polymerization or (2) be captured by functionalized nitroxide-based radical traps in the MILRad functionalization approach. For the second case, terminal α-bromoisobutyrate was installed at the polyolefin chain, which is essential for block copolymerization. However, the functionalization efficiency of the radical trapping by the TEMPO ((2,2,6,6-tetramethylpiperidin-1-yl)oxyl)-based radical traps was not quantitative.^63^

Unlike the MILRad strategy, the polyolefin active-ester exchange (PACE) approach^59^ does not rely on a homolytic cleavage of the Pd–C bond and subsequent radical trapping to functionalize a growing chain end. Instead, an active ester moiety served as an integral part of the late transition complex. Upon olefin polymerization, the functionalized single-chain end terminus became a useful handle for efficient postpolymerization reactions. The pentafluorophenyl (PFPh) ester-chelated palladium catalyst afforded a valuable chain-end functionalization but also supported the productivity and livingness of the olefin polymerization.^59,64,66^

Capitalizing on the highly reactive single-chain end functionality, we introduced in this work the highly electron-withdrawing hexafluoroisopropyl (HFIP) ester moiety as an alternative to the PFPh group. The advantages of fluorinated ester are well documented; the isopropyl derivative is anticipated to enforce high yielding transformations, while a highly volatile hexafluoroisopropanol can be easily removed from the reaction mixtures. Furthermore, the livingness of the ethylene polymerization and the ease of transesterification processes with HOBIB and HOBIBA allow for facile access to polyolefin-based ATRP macroinitiators. Using such initiators, polar polyolefin block copolymers were easily synthesized via SARA ATRP, as demonstrated with methyl acrylate as the model acrylate monomer. Figure 1 illustrates the synthetic pathways to PE macroinitiators for ATRP using both the reported earlier MILRad functionalization and the PACE approach presented in this study.

## 2 Results and Discussion

### 2.1 Synthesis of Hexafluoroisopropyl-Ester Terminated Polyethylene (PE-HFIP)

**Figure 2:**
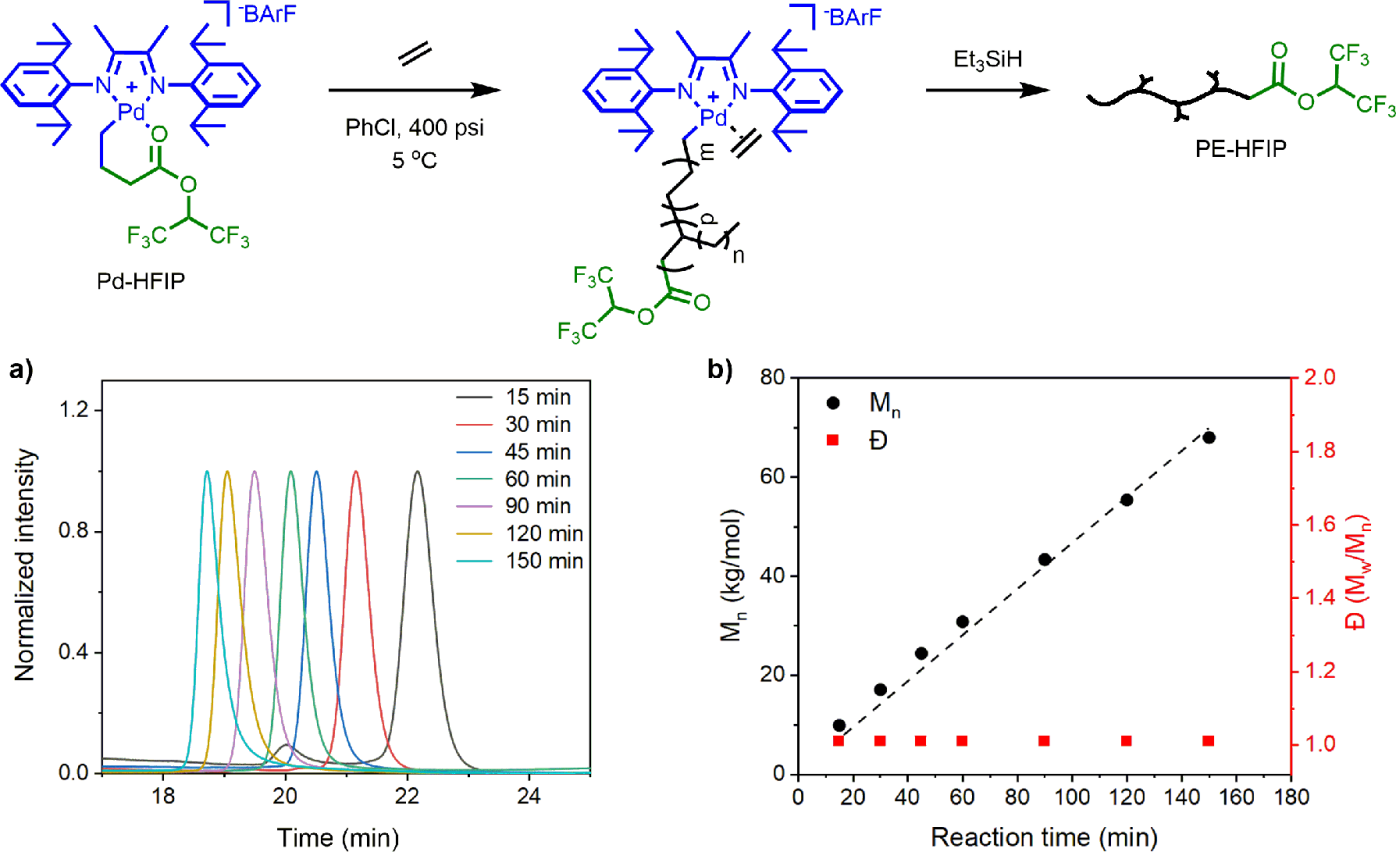
Kinetic study of PE homopolymerization: (a) GPC traces of PE homopolymers for different reaction times in THF (Table S1) and (b) *M*_n_ vs reaction time plot confirmed living polymerization of ethylene.

We commenced with the synthesis of PE-HFIP from the known diimine Pd(II) Brookhart-type catalyst precursor (precatalyst 1A, Figure S1) by chelating hexafluoroisopropyl acrylate as outlined in S1 and S2. This process formed the Pd-HFIP complex, the hexafluoroisopropyl ester diimine Pd(II) macrochelate (Figures 1 and 2). This Pd-HFIP complex was analyzed by ^1^H NMR (Figure S3), ^13^C NMR (Figure S4), and ^19^F NMR (Figure S5), and a crystal structure was also obtained through X-ray diffraction analysis (Figure S6).

We sought to investigate if the nature of electron-withdrawing fluorinated substituent influences the activity of the catalyst complex and the livingness of ethylene polymerization. Previously, we reported that a pentafluorophenyl substituent in Pd-PFPh overcame issues of known acrylate-chelated Pd(II) diimine complexes, which showed a limited activity.^59^

A kinetic study of ethylene polymerization with Pd-HFIP was performed by varying the reaction time to investigate the livingness window of the ethylene homopolymerization (Figure 2 and Table S1). Figure 2 indicated that a high level of control over the polymerization was achieved via Pd-HFIP, comparable to that of Pd-PFPh. A linear correlation between reaction time and molecular weight (*M*_n_) was observed that followed the first-order kinetics from 15 to 150 min. The *M*_n_ increased from 10 to 68 kg/mol while maintaining a low dispersity (*Đ* ≈ 1.01). Moreover, a higher level of catalytic activity was observed compared to the previously utilized Pd-PFPh; the turnover frequency (TOF) of Pd-HFIP was 1,000 whereas that of Pd-PFPh was 770 (Figure S7).

### 2.2 Ester-Linked PACE-Prepared Macroinitiator (PE-EBiB-MI)

The PE-HFIP readily underwent transesterification with 2-hydroxyethyl α-bromoisobutyrate, resulting in the installation of ATRP-initiable functionality to the polyethylene chain (Figure S8). This transesterification yielded the polyethylene-based macroinitiator terminated with α-bromoisobutyrate, heretofore termed PE-EBiB-MI. This result illustrated how the PACE mechanism can be utilized to prepare PE macroinitiators for ATRP.

#### 2.2.1 Model SARA ATRP Reactions

The model reactions confirmed successful syntheses of poly(methyl acrylate) homopolymer in chlorobenzene using HOBIB as the initiator analog. Chlorobenzene was selected as the solvent, as it dissolved both the polyolefin and polyacrylate blocks. The dielectric constant of chlorobenzene is 5.62, which is greatly lower than that of common ATRP solvents such as dimethylformamide (DMF, 36.7) and dimethyl sulfoxide (DMSO, 46.7). Thus, the lower polarity of chlorobenzene resulted in lower value of *K*_ATRP_ and slower rate of polymerization.^67,68^ Despite such drawbacks, chlorobenzene was selected as the reaction medium since it was previously used as the solvent to successfully carry out block copolymerization of PE-*b*-PMA via MILRad.^63^

SARA ATRP,^69^ supplemental activator and reducing agent atom transfer radical polymerization, was employed as the block copolymerization technique. SARA ATRP, employing metallic copper wire, previously showed well-controlled polymerization and high levels of initiation efficiencies.^63^

Furthermore, under the SARA ATRP approach, Cu(II) was reduced to Cu(I) via comproportionation with metallic Cu^0^. Hence, no additional radical species are generated during the catalyst reduction process, unlike the other regenerative low-ppm ATRP approaches. Other regenerative ATRP approaches, such as ICAR ATRP (initiators for continuous activator regeneration ATRP), utilized radical initiators such as azo compounds, which may undergo propagation to form polyacrylate homopolymers.^70^ By use of SARA ATRP, such side reactions can be successfully suppressed to achieve the maximum blocking efficiencies.

SARA ATRP involves the comproportionation (*k*_comp_) of Me_6_Tren/CuBr_2_ and Cu(0) wire to form Me_6_Tren/CuBr as the activator complex species (Me_6_Tren:tris[2-(dimethylamino)ethyl]amine). Hence, the concentration of the radical species for SARA ATRP is proportional to the concentration of CuBr_2_ and the Cu^0^ wire surface area. Alkyl halide initiators can also directly react with Cu(0) and form radicals via supplemental activation (*k*_SA_). Rates of both heterogeneous reactions scale with the ratio of the surface area of the copper wire (*S*) to the reaction volume (*V*). The steady state of radical concentration was established via both comproportionation and supplemental activation vs radical termination, according to the earlier mechanistic studies on SARA ATRP:^71,72^
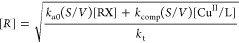
1

The concentration of radicals defines the kinetics of ATRP. As shown in eq 2, the rate of polymerization depends on the concentration of monomers (*M*), the propagation rate constant (*k*_p_*),* and the radical concentration ().
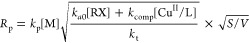
2

**Figure 3:**
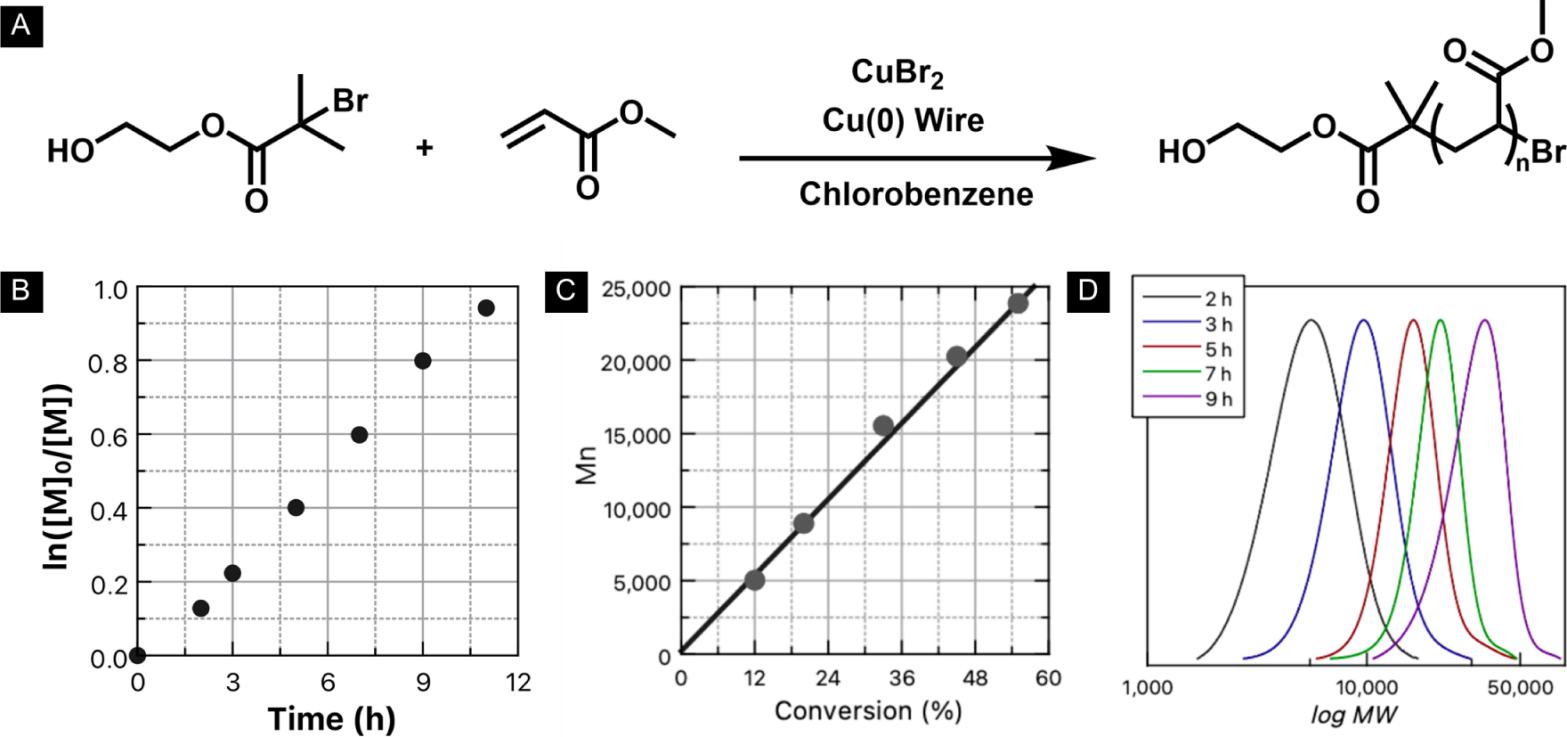
Poly(methyl acrylate) synthesized with 2-hydroxyethyl α-bromoisobutyrate initiator via SARA ATRP. (A) Synthesis scheme, (B) dependence of ln([M]_0_/[M]) on time, (C) dependence of molecular weight on conversion, and (D) THF GPC traces at time = 2 to 9 h. Molar ratios: [HOBIB]:[MA]:[CuBr_2_]:[Me_6_Tren] = 1:500:0.04:0.1. Cu^0^ wire surface area = 0.32 cm^2^. [MA]_0_ = 5.5M, chlorobenzene as solvent (45 vol %) at room temperature. All reagents were deoxygenated with N_2_ prior to polymerization. Total reaction volume: 2.20 mL.

First, poly(methyl acrylate) was synthesized with HOBIB in chlorobenzene, as shown in Figure 3A. The detailed procedure of the SARA ATRP is outlined in Figure S9. A linear semilogarithmic relationship between conversion and time is shown in Figure 3B. Furthermore, a close match between the theoretical and experimental molecular weight indicated efficient and controlled polymerization (Figure 3C). Finally, a gradual shift of the unimodal peak from low to high molecular weight was observed in the GPC traces (Figure 3D).

**Figure 4:**
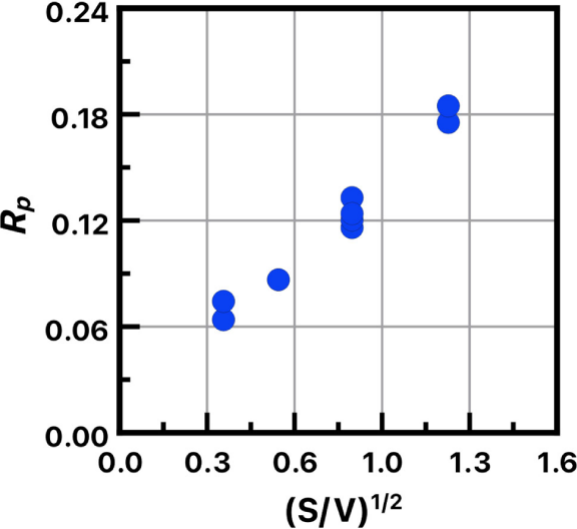
Poly(methyl acrylate) synthesis via SARA ATRP. Dependence of the rate of polymerization on (*S*/*V*)^1/2^; Molar ratios: [HOBIB]:[MA]:[CuBr_2_]:[Me_6_Tren] = 1:500:0.04:0.1. Cu^0^ wire surface area = 0.32 cm^2^. All reagents deoxygenated with N_2_ prior to polymerization, chlorobenzene as solvent at room temperature.

Figure 4 illustrates the kinetics of SARA ATRP of methyl acrylate in chlorobenzene with HOBIB as the model initiator and Me_6_Tren as the ligand. Polymerization reactions were conducted at various *S*/*V* ratios. The rates of SARA ATRP scale linearly with the *S*/*V*^1/2^ values (eq 2). Successful polymerizations were conducted at various wire surfaces and volumes down to *V* = 0.45 mL.

#### 2.2.2 Characterization of the Macroinitiator, PE-EBiB-MI

The polyolefin macroinitiator was prepared via the PACE approach. The polyethylene chain, prepared with a Pd catalyst via coordination–insertion polymerization, underwent quenching via triethylsilane and transesterification to prepare ester-linked macroinitiators. This macroinitiator, terminated with α-bromoisobutyrate functionality, is referred to as *PE-EBiB-MI*.

**Figure 5:**
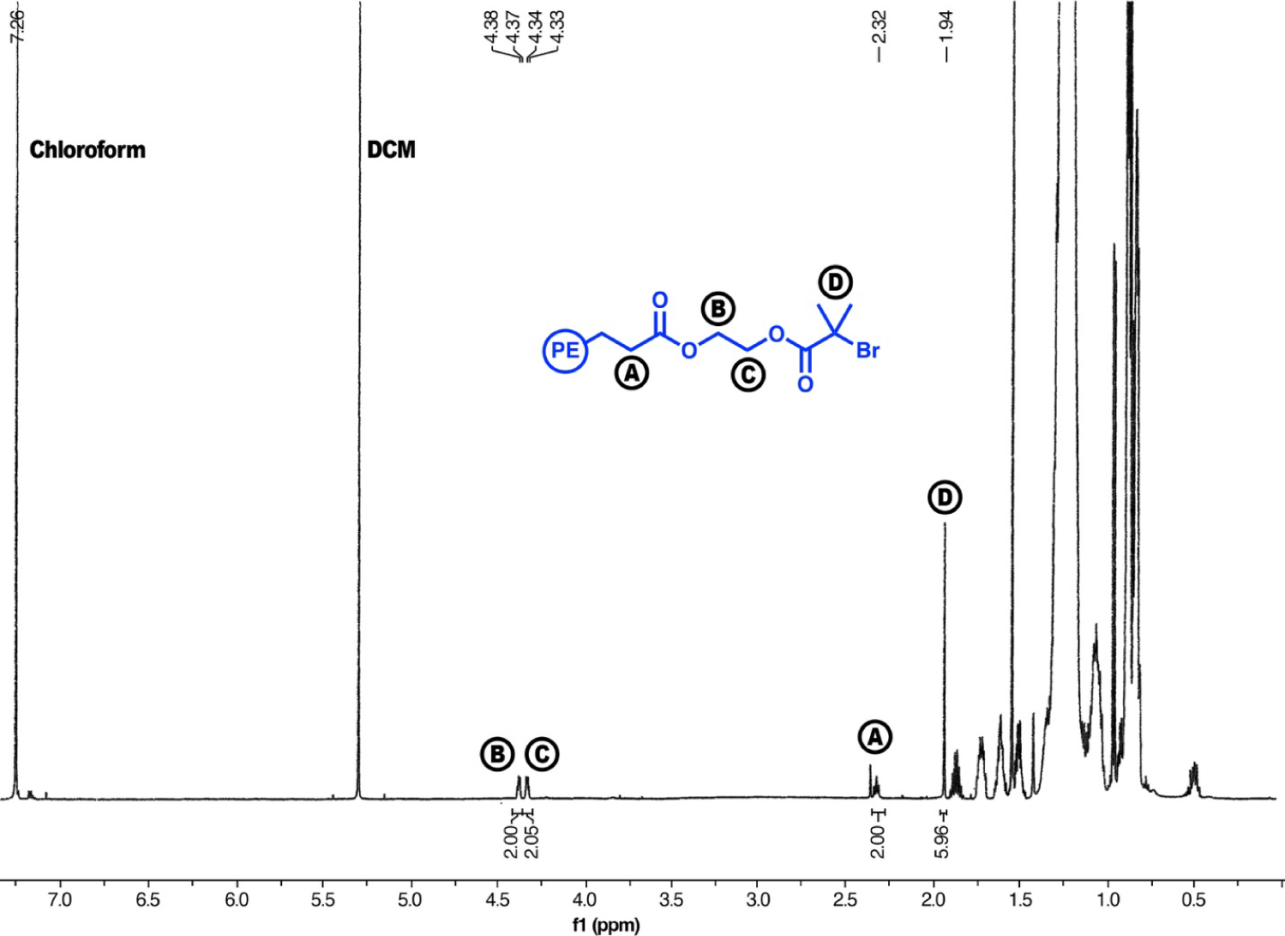
^1^H NMR spectra (scan numbers = 128, CDCl_3_, 25 °C, 400 MHz) of the ester-linked macroinitiator, *Es-MI-01*, postfunctionalization and purification. Excess HOBIB was removed via dissolving the concentrated polymer in a minimal amount of hexane, followed by centrifugation and the removal of excess triazabicyclo[4.4.0]dec-5-ene (TBD).


The *PE-EBiB-MI* sample utilized for the block copolymerization, *Es-MI-01*, was analyzed via ^1^H NMR spectroscopy, as shown in Figure 5. The integration of the α-methylene protons adjacent to the carbonyl functional group (**A**) was set to 2. The methylene protons (**B, C**) were integrated to 2.00 and 2.05. The terminal protons (**D**) were integrated to 5.96. The chain-end functionality of the macroinitiator was 99.3%. This was calculated by dividing the terminal protons by the proton adjacent to the carbonyl functional group, as shown in eq 3.

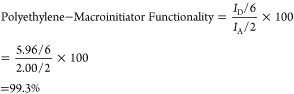
3

#### 2.2.3 Block Copolymerization by SARA ATRP

**Figure 6:**
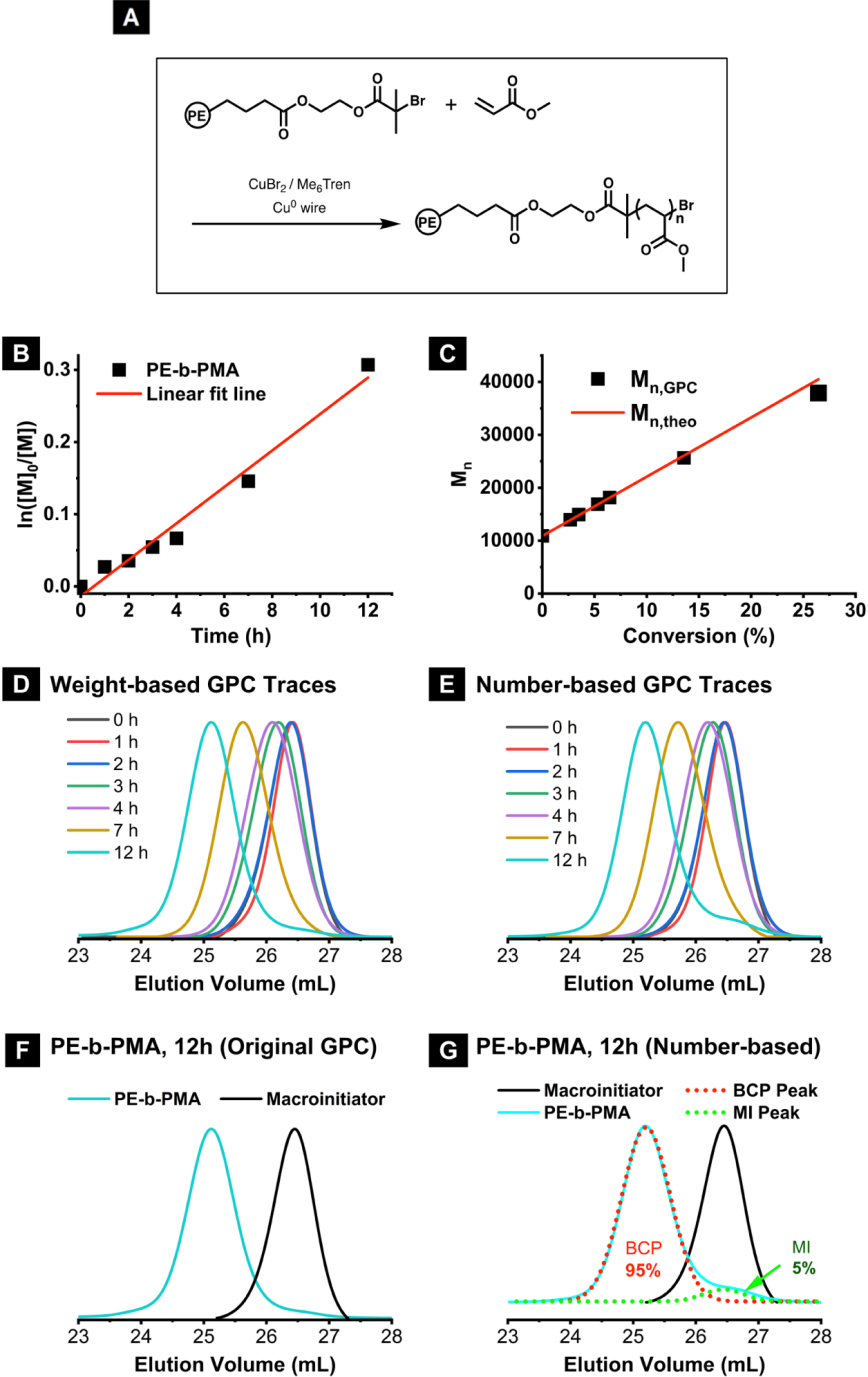
PE-*b*-PMA synthesized with Es-MI-01. (A) Synthetic scheme, (B) dependence of ln([M]_0_/[M]) on time, (C) dependence of molecular weight on conversion, (D) THF GPC traces, weight-based, (E) number-based GPC traces, (F) THF GPC traces of PE-*b-*PMA BCP and the macroinitiator, and (G) chain-number based distribution. Blue solid line: PE-*b-*PMA BCP. Red dotted line: deconvoluted BCP peak. Green dotted line: deconvoluted MI peaks. Black solid line: macroinitiator peak. Molar ratio: [Macroinitiator]:[MA]:[CuBr_2_]:[Me_6_Tren] = 1:1300:0.1:0.6. Cu^0^ wire (0.5 mm diameter, 6 cm, surface area = 0.95 cm^2^) was used. Reaction at room temperature. [MA]_0_ = 4.5 M, 60 vol % chlorobenzene. [CuBr_2_] = 75 ppm.

The synthesis of polyethylene-*b*-PMA BCPs using ester-linked macroinitiators via SARA ATRP is illustrated in Figure 6A. The detailed procedure of the block copolymerization is outlined in Figure S10. The polymerization process exhibited good control, as evidenced by the data presented in Figure 6B,C. A linear semilogarithmic relationship between the monomer conversion time and the conversion time was observed. A close agreement between the theoretical and experimental molecular weights indicated the effective initiation of the macroinitiators.

In Figure 6D, the GPC traces showed a gradual increase in the molecular weight of the BCP over time, with minimal detection of uninitiated macroinitiators. The PE-*b-*PMA block copolymer was further analyzed via ^1^H DOSY (diffusion-ordered spectroscopy) (Figure S11). The obtained signals confirmed that the PE block and the PMA block are covalently bonded.

To convert the original GPC traces to the number-based GPC traces, the refractive index (RI) was divided by the calibrated molecular weight (cf. example shown in Figure S12). The resulting number-based GPC traces, as shown in Figure 6E, revealed a slight residual amount of the macroinitiator remaining at an elution volume of approximately 26.5 mL. The residual peak corresponded to the macroinitiator measured at 0 h. This suggested the presence of a small amount of the macroinitiator that was not activated for the block extension.

The sample following the second block extension for 12 h is plotted in Figure 6F. Based on the original THF GPC traces, no significant amount of residual macroinitiator was observed. The number-based GPC traces were deconvoluted to fit two Gaussian peaks to calculate the relative ratio of chain number for block copolymer and macroinitiator. The deconvoluted traces, shown in Figure 6G, indicated that approximately 95% of the total chains were BCP chains, with the remaining 5% macroinitiator. This suggested that a nearly quantitative initiation of the macroinitiator was achieved under these conditions.

The PE-*b*-PMA block copolymers were prepared via the SARA approach by using PACE-prepared macroinitiators. The results are summarized in Table 1. The macroinitiator had *M*_n_ = 12,100 and a dispersity of 1.01. Entry 1 indicated that the polymerization process did not proceed without copper wire. Polymerization required comproportionation to form copper(I) species, following the SARA pathway. With copper wire, a 10% conversion was achieved within 1 h. An agreement between the theoretical and experimental molecular weights was observed, as determined by the ^1^H NMR and the GPC analyses, respectively. A low dispersity of 1.09 was achieved, indicating well-controlled polymerizations.

**Table 1:** PE-*b*-PMA Block Copolymers Prepared via the PACE-SARA Approach^a^

#	[M]_0_	Reaction Vol. (mL)	Time (h)	Wire length (cm)	Cu^0^ Surface Area (cm^2^)	MA Conv. (%)	*M*_n,th_	*M*_n_	*Đ*_BCP_
1	5.05	0.44	4	0	0.00	0	12,100	-	-
2^b^	5.05	0.44	4	0 → 2	0.00 → 0.32	15	17,200	13,400	1.03
3	5.05	0.44	2	1	0.16	17	18,000	21,600	1.07
4	5.05	0.44	4	1	0.16	24	20,400	20,700	1.13
5	5.05	0.44	4	1	0.16	24	20,400	19,300	1.14
6	5.05	0.44	4	1	0.16	23	20,000	23,300	1.12
7	5.05	0.44	1	2	0.32	10	15,500	14,900	1.09
8	5.05	0.44	2	2	0.32	22	19,700	23,300	1.03
9	5.05	0.44	4	2	0.32	36	27,600	26,900	1.03

a Ester-linked macroinitiator: *M*_n_ = 12,100, *Đ* = 1.01. Molar ratios: [Macroinitiator]:[MA]:[CuBr_2_]: [Me_6_Tren] = 1:500:0.04:0.1. All reagents were deoxygenated with N_2_ prior to block copolymerization. Chlorobenzene (45 vol %) at room temperature. Total reaction volume = 0.44 mL

b MA, methyl acrylate, was the monomer for the second block using SARA ATRP. a) Entry 2, Cu^0^ wire added at *t* = 2 h. Cu^0^ wire (0.5 mm diameter, 2 cm, Surface area = 0.32 cm^2^) added. SARA ATRP: Supplemental activator and reducing agent atom transfer radical polymerization. PE: Polyethylene. PMA: Poly(methyl acrylate). PACE: Polyolefin active ester exchange. MA: Methyl acrylate. HOBIB: 2-Hydroxyethyl α-bromoisobutyrate.

For entry 2, no copper wire was added at the initial stage of the reaction. After 2 h, 2 cm of copper wire was introduced to the reaction mixture under continuous N_2_ injection to maintain an oxygen-free environment. A noticeable change in viscosity was observed following the addition of the copper wire, resulting in a final conversion of 15% and a low dispersity of 1.03.

When 1 cm of copper wire was used, a 17% conversion was reached at a reaction time of 2 h. The experimental molecular weight closely matched the theoretical value, and a low dispersity of 1.07 was observed (Entry 3). For entries 4 to 6, identical experimental conditions were used to ensure the reproducibility of the experiment. The conversions for the three samples were 24%, 24%, and 23%. The dispersities ranged from 1.12 to 1.14. The proximity of the polymers indicated high reproducibility of the reaction.

Entries 7–9 show the polymerization results after 1 to 4 h. The conversion increased from 10% to 36%, from 1 to 4 h. For all samples from entries 7 to 9, a good agreement between the theoretical and experimental molecular weight was observed, along with low dispersity (*Đ* < 1.10). The good match between the theoretical and experimental molecular weight showed a high initiation efficiency, which together with the low dispersity indicated a highly controlled polymerization process.

### 2.3 Amide-Linked PACE-Prepared Macroinitiator (PE-EBiBA-MI)

As discussed above, block copolymers were successfully synthesized by using ester-linked macroinitiators. To test the effects of initiator chain-end structure, amide-linked tertiary-bromide-capped initiators were used for the synthesis of PMA.

#### 2.3.1 Model SARA ATRP Reactions

HOBIBA was used as the amide-linked initiator analog to synthesize PMA in chlorobenzene, and the results of the homopolymerization by SARA ATRP are summarized in Table 2.

**Table 2:** Poly(methyl acrylate) Homopolymers Prepared from SARA ATRP^a^^b^

#	Reaction Vol. (mL)	Initiator	Time (h)	Wire length (cm)	Cu^0^ Surface Area (cm^2^)	MA Conv. (%)	*M*_n,th_	*M*_n_	*Đ*
1	0.44	HOBIB	2	2	0.32	22	18,400	18,200	1.04
2	0.44		3	2	0.32	27	23,000	25,000	1.07
3	0.44	HOBIBA	2	2	0.32	26	11,400	11,300	1.06
4	2.20		3	2	0.32	31	13,500	13,400	1.19

a MA, methyl acrylate, was the monomer for the polymerization. SARA ATRP: Supplemental activator and reducing agent atom transfer radical polymerization. HOBIB: 2-Hydroxyethyl α-bromoisobutyrate. HOBIBA: 2-Hydroxyethyl α-bromoisobutyramide.

b Molar ratios: [Initiator]:[MA]:[CuBr_2_]:[Me_6_Tren] = 1:500:0.04:0.1. Cu^0^ wire surface area = 0.32 cm^2^. All reagents were deoxygenated with N_2_ prior to polymerization. Chlorobenzene (45 vol %) at room temperature.

Entries 1 and 2 relate to the homopolymers synthesized from ester-containing HOBIB. The conversion reached 42% and 52% after 2 and 3 h, respectively, with a close match between theoretical and experimental molecular weights and low dispersity.

Entries 3 and 4 relate to the homopolymers synthesized from amide-containing HOBIBA. In entry 3, the HOBIBA-initiated PMAs had a low dispersity of 1.06. In entry 4, a successful polymerization with a dispersity below 1.20 was shown. A higher reaction volume of 2.20 mL was employed, resulting in a slower conversion rate of 31% after 3 h. This was due to a lower surface-to-volume ratio (S/V). Additionally, for both entries, the theoretical and experimental molecular weights were well-matched. These results indicated that amide-linked initiators were also successfully utilized in the SARA ATRP in chlorobenzene.

**Figure 7:**
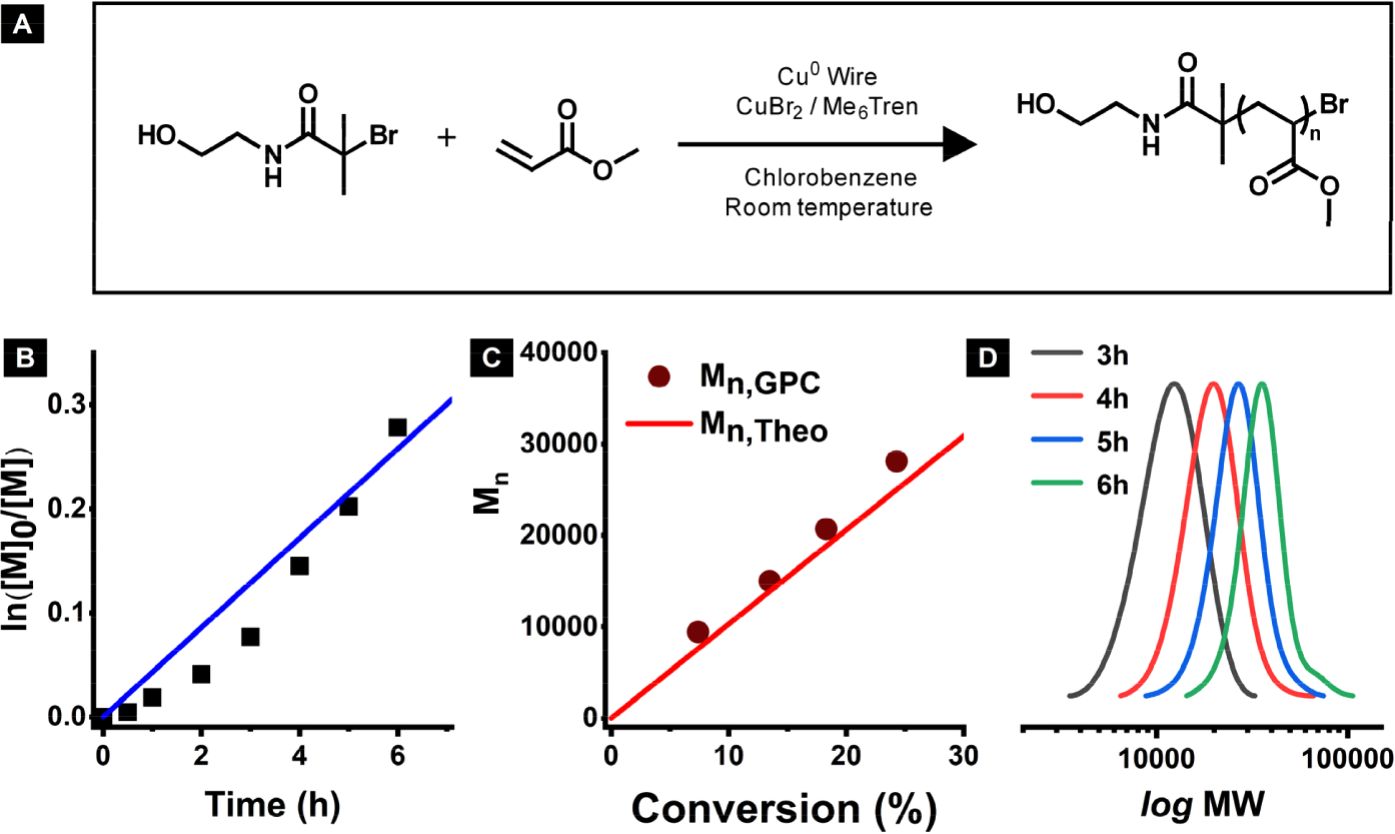
Poly(methyl acrylate) synthesized using 2-hydroxyethyl α-bromoisobutyramide (HOBIBA) via SARA ATRP. (A) Synthesis scheme, (B) dependence of ln([M]_0_/[M]) on time, (C) dependence of molecular weight on conversion, and (D) THF GPC traces at time = 3 to 6 h. Molar Ratios: [HOBIBA]:[MA]:[CuBr_2_]:[Me_6_Tren] = 1:1300:0.1:0.6. Cu^0^ wire (0.5 mm diameter, 6 cm, surface area = 0.95 cm^2^) was used as the reducing agent. [MA]_0_ = 5.5M, 45 vol % chlorobenzene at room temperature. All reagents were deoxygenated with N_2_ prior to polymerization.

The polymers synthesized with HOBIBA were further studied via kinetic experiments, as shown in Figure 7. A linear semilogarithmic relationship between conversion and time is shown in Figure 7B. A short induction period was observed. A close match between the theoretical and the experimental molecular weight was observed as demonstrated in Figure 7C. Finally, the GPC traces showed a gradual unimodal peak shift from low to high molecular weight while maintaining low dispersity, showing controlled and successful polymerization of PMA in chlorobenzene via SARA ATRP over a 3 to 6 h period (Figure 7D).

Following the successful synthesis of HOBIBA-prepared PMA, amide-linked macroinitiators were prepared. As shown in Figure S13, the transesterification of HOBIBA with the PE-HFIP resulted in the formation of the PE-based macroinitiator, terminated with α-bromoisobutyramide functionality, referred to as PE-EBiBA-MI. Following the similar procedure used for the analysis of the ester-based macroinitiators, proton NMR and GPC were used to characterize the macroinitiator, with *M*_n_ = 12,000 and *Đ* = 1.01. The functionalization efficiency of the amide-based macroinitiator was 93%, possibly due to the elimination reaction following an E2 mechanism (Figure S14), caused by the presence of a strong base (triazabicyclo[4.4.0]dec-5-ene, TBD) and a good leaving group (bromide).

**Figure 8:**
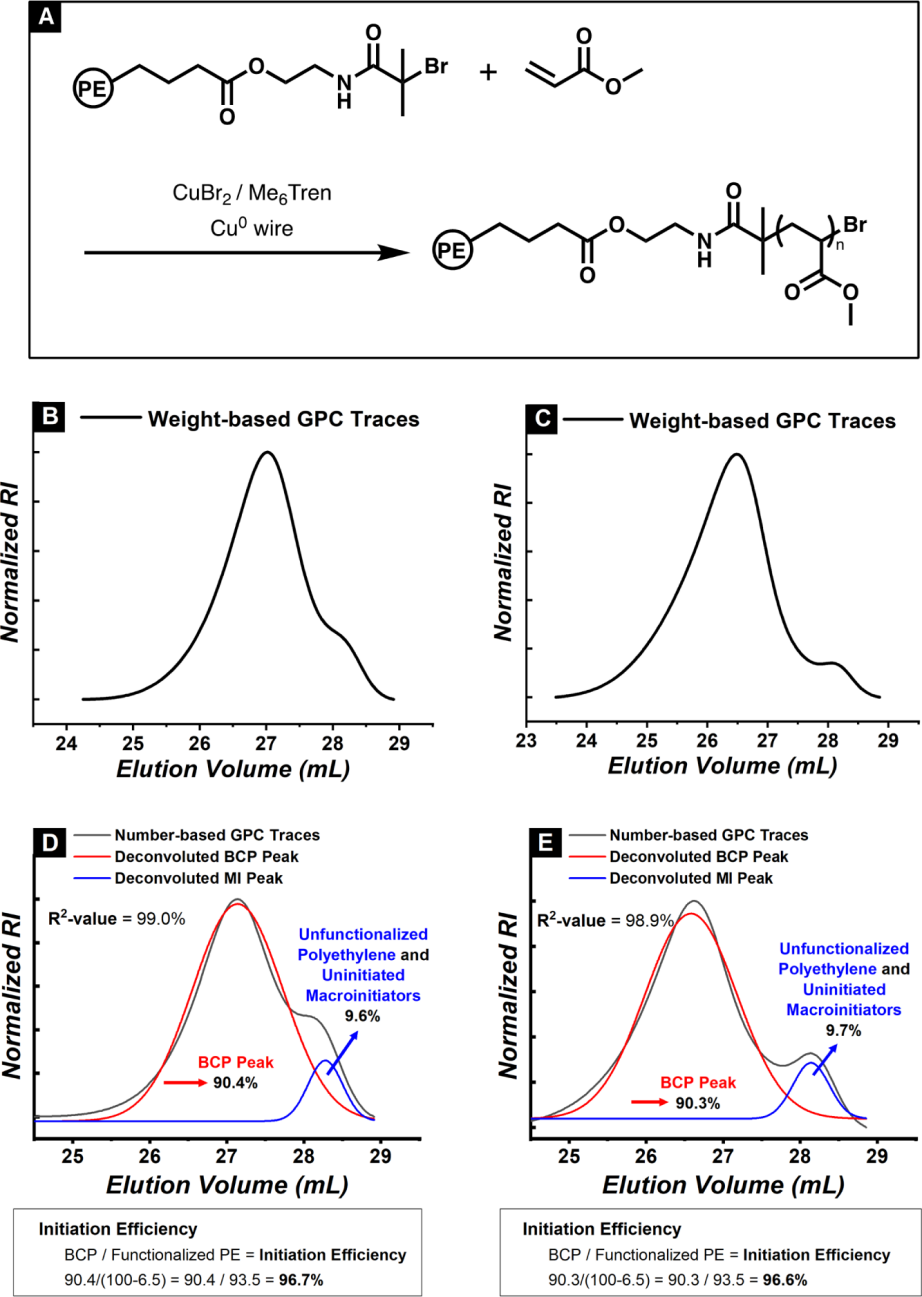
GPC and deconvoluted peak traces of PE-*b*-PMA prepared via the PACE-SARA ATRP approach. PE-EBiBA-MI used for the block copolymerization: *M*_n_ = 12,000, *Đ* = 1.01, percent functionalization = 93.5%. (A) Synthetic scheme of PE-*b*-PMA, (B) weight-based GPC chromatogram, experimental condition A, (C) weight-based GPC chromatogram, experimental condition B, (D) number-based GPC traces and deconvoluted peaks traces of the PE-*b*-PMA, experimental condition A, and (E) number-based GPC traces and deconvoluted peak traces of the PE-*b*-PMA, experimental condition B. *General reaction condition:* [MA]_0_ = 5.05 M, Total reaction volume = 0.88 mL, Molar ratios: [Macroinitiator]:[CuBr_2_]:[Me_6_Tren] = 1:0.04:0.1, Cu^0^ wire surface area = 1.57 cm^2^. All of the reagents were deoxygenated with N_2_ prior to block copolymerization. *Condition A)* [Macroinitiator]:[MA] = 1:500, Chlorobenzene (45 vol %) and dimethylformamide (9 vol %) as solvent. Reaction time: 6.5 h. *Condition B)* [Macroinitiator]:[MA] = 1:700, Chlorobenzene (34 vol %) and dimethylformamide (20 vol %) as solvent. Reaction time: 6 h.

Based on the results involving HOBIBA, BCPs prepared by using amide-linked macroinitiators were anticipated to have a high level of initiation. Figure 8A shows the block copolymer synthesis scheme, initiated with amide-linked macroinitiators. The original GPC traces (Figure 8B,C) were converted to a chain-number distribution, followed by deconvolution, as shown in Figure S12. In Figure 8D, a residual macroinitiator peak was observed. The major peak associated with block copolymers (red line) was integrated to 90.4%. The minor peak (blue line) was integrated to 9.6%. The minor peak reflected both the unfunctionalized polyethylene chains (6.5%) and uninitiated macroinitiators (3.1%). The initiation efficiency was calculated by dividing the ratio of the block copolymer chain (90.4%) by the sum of the block copolymer chain and uninitiated macroinitiators (93.5%). The final initiation efficiency of the sample analyzed in Figure 8D was 96.7% (eq 4).
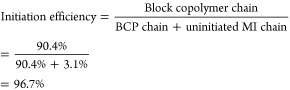
4

Similarly, the residual macroinitiator peak was observed in Figure 8E. The major peak associated with block copolymers (red line) was integrated to 90.3%. The minor peak (blue line) was integrated to 9.7%. The final initiation efficiency of the sample analyzed in Figure 8D was 96.6%. Despite different solvent compositions and reaction times, near-quantitative initiation efficiencies were observed when the key factors were constant.

In Table 3, PE-EBiBA-MI was used to prepare PE-*b*-PMA block copolymers. Initially, the initiation efficiency was limited to under 75%. (entries 1, 2). However, with the increase in the S/V ratio, the initiation efficiency increased to 97% in entries 4, 5.

**Table 3:** PE-*b*-PMA Block Copolymers Prepared via the PACE-SARA ATRP Approach^a^

#	[M]_0_	Reaction Volume (mL)	Time (h)	Wire length (cm)	Cu^0^ Surface Area (cm^2^)	MA Conv. (%)	*M*_n,th_	*M*_n_	*Đ*_BCP_	Init. Eff. (%)
1	5.05	0.44	7	2	0.32	45	31,400	42,900	1.05	72
2	3.37	0.66	8	4	0.63	25	22,800	33,500	1.03	73
3	3.37	0.66	8	8	1.26	36	27,500	35,800	1.07	85
4	5.05	0.88	6.5	10	1.57	40	29,400	25,800	1.15	97
5^b^	5.05	0.88	6	10	1.57	50	33,700	31,300	1.22	97

a Amide-linked macroinitiator PE-EBiBA-MI was used for the block copolymerization: *M*_n_ = 12,000, *Đ* = 1.01. Molar ratios: [Macroinitiator]:[MA]:[CuBr_2_]:[Me_6_Tren] = 1:500:0.04:0.1. All reagents deoxygenated with N_2_ prior to block copolymerization. Chlorobenzene (45 vol %) at room temperature. Approximately 9 vol % of dimethylformamide in each reaction.

b MA, methyl acrylate, was the monomer for the second block using SARA ATRP. a) Entry 5, approximately 20% of dimethylformamide in the reaction solution. Molar Ratios: [Macroinitiator]:[MA]:[CuBr_2_]:[Me_6_Tren] = 1:700:0.04:0.1. SARA ATRP: Supplemental activator and reducing agent atom transfer radical polymerization. PE: Polyethylene. PMA: Poly(methyl acrylate). PACE: Polyolefin Active Ester Exchange.

The increase in the initiation efficiency with an increase in the S/V ratio may be related to incomplete O_2_ degassing. Due to the highly unstable nature of the radicals, it is important to remove all radical-scavenging oxygen molecules before the ATRP polymerization. Faster generation of copper(I) via comproportionation with an increased S/V ratio minimized such negative impacts caused by the remnant oxygen in the reaction vial.

Entry 1 showed that the initiation efficiency reached 72% after 7 h of polymerization. Initiation efficiency was calculated by converting the weight-based GPC traces into the number-based GPC traces, followed by a deconvolution (Figure S12). Approximately 28% of the macroinitiator was not initiated for the second block extension. Furthermore, the experimental molecular weights were higher than the theoretical molecular weights for both entries, which implied limited initiation efficiencies of the amide-based macroinitiators.

In entry 2, the monomer concentration was reduced to 3.37 M. This adjustment did not significantly impact the initiation efficiency, which remained at 73%. The experimental molecular weight was also higher than the theoretical molecular weight. However, the conversion was lower compared to that in the previous entry due to the lower monomer concentration. For entry 3, the surface of the copper wire was doubled to 1.26 cm^2^, increasing the (*S*/*V*)^1/2^ ratio to 1.38. This change led to a 36% conversion after 8 h and an increase in the initiation efficiency up to 85%. An increase in the copper wire surface area is correlated with enhanced initiation efficiency.

For entry 4, conversion reached 40% after 6.5 h. A good match between the theoretical and experimental *M*_n_ was observed with a low dispersity of 1.15. The initiation efficiency reached 97%. In entry 5, dimethylformamide (DMF) v/v% increased from 9% to 20%, and the monomer-to-initiator ratio was increased from 500:1 to 700:1. The conversion accelerated and reached 50% at 6 h and the initiation efficiency also reached 97%. The series of optimizations in Table 3 demonstrated that higher initiation efficiency was achieved with (1) increased monomer concentration and (2) increased ratio of *S*/*V*.

## 3 Conclusions

In summary, we present the facile PACE-SARA ATRP method to prepare polyolefin-polyacrylate block copolymers by fusing two living polymerization methods. Hexafluoroisopropyl acrylate-chelated Pd(II) diimine complexes exhibited a higher catalytic activity than the previously investigated pentafluorophenyl analogs with a preserved livingness over ethylene polymerizations. Transesterification with either 2-hydroxyethyl α-bromoisobutyrate or 2-hydroxyethyl α-bromoisobutyramide resulted in the desired polyolefin macroinitiators in high yield.

Both macroinitiators, ester-linked PE-EBiB-MI and amide-linked PE-EBiBA-MI, exhibited 95% and 97% initiation efficiency in SARA ATRP, respectively. Low dispersity of the block copolymers confirmed well-controlled polymerizations with high degrees of chain-end livingness. The facile introduction of ATRP initiators to functionalized PE chains opened up access to a wide variety of hybrid polyolefin-polar acrylic architectures.

## Supporting Information Available

Various synthetic procedures, schemes, and details on the homopolymer/block copolymer synthesis (PDF)
Crystal structure of the Pd-HFIP ester compound (CIF)
Crystal data and structure refinement for 2A-HFIPA_XW626, etc. (PDF)

## Supplementary Material

ma4c02684_si_001.pdf

ma4c02684_si_002.cif
