# Supplementary material for: Polyethylene-poly(methyl acrylate) Block Copolymers from PACE-SARA ATRP: Utilizing Polyolefin Active Ester Exchange-Based Macroinitiators in Atom Transfer Radical Polymerization
Source: Macromolecules. 2025 Jan 30;58(3):1337–48. doi: 10.1021/acs.macromol.4c02684 (PMC11823591; doi:10.1021/acs.macromol.4c02684)
Supplement: Supplementary file 1 — ma4c02684_si_001.pdf [file ma4c02684_si_001.pdf]

Supporting Information

Polyethylene-poly(methyl acrylate) Block Copolymers from PACE-SARA ATRP: Utilizing Polyolefin Active Ester Exchange-Based Macroinitiators in Atom Transfer Radical Polymerization.

Khidong Kim[a], Jacobo Strong[b], Stephen Don Sarkar[b], Dung Nguyen[b], Huong Dau[b], D.A. Anwar Al-Aman[b], Sajjad Dadashi-Silab[a], Eva Harth[b] and Krzysztof Matyjaszewski[a]**

[a] Department of Chemistry, Carnegie Mellon University, Pittsburgh, PA, 15213, USA

[b] Department of Chemistry, Center of Excellence in Polymer Chemistry (CEPC), University of Houston, Houston, 77204 Texas, USA

** E-mail: harth@uh.edu*

** E-mail: km3b@andrew.cmu.edu*

Table of Contents

1. Materials *3*

1.1 Materials: Macroinitiator Synthesis 3

1.2 Materials: Block Copolymer Synthesis 3

1.3 Instrumentation (Macroinitiator Synthesis) 4

1.4 Instrumentation (Block Copolymer Synthesis) 5

2. Procedures for Macroinitiator Synthesis *6*

2.1 Ligands and Catalysts 6

2.2 Macroinitiator Synthesis 13

3. Ester-linked tertiary-bromide capped initiators *17*

3.1 PMA Homopolymer synthesis via SARA ATRP 17

3.2 PE-*b*-PMA Block Copolymer Synthesis via SARA ATRP 18

3.3 Block copolymer PE-*b*-PMA 2D NMR Results (Diffusion-ordered spectroscopy, DOSY) . 19

4. Calculation method for block copolymer initiation efficiency *19*

5. Amide-linked tertiary-bromide capped initiators *22*

5.1 Amide-linked PACE-prepared MI synthesis 22

5.2 Elimination side reaction for amide linked MI 23

1. Materials

1.1 Materials: Macroinitiator Synthesis

Calcium hydride powder (CaH_2, ≥ 90%), diethyl ether anhydrous (≥ 99.7%), acetonitrile ACS reagent (≥ 99.5%), toluene (HPLC, 99.9%), 2,3-butanedione (97%), MgSO4 (≥ 99.0%), chlorobenzene(anhydrous, 99.8%), 1,1,1,3,3,3-hexafluoroisopropyl acrylate (99%), ethanolamine ACS reagent (≥ 99.0%), ethylene glycol anhydrous (99.8%), α-bromoisobutyryl bromide (98.0%), and triazabicyclo[4.4.0]dec-5-ene (98%) were purchased from Sigma-Aldrich and used as received unless otherwise noted. 2,6-Diisopropylaniline (90%) was purchased from Oakwood Chemical and used as received. Formic acid (≥ 98%) was purchased from Millipore Sigma and used as received. Methanol (99%), methylene chloride (DCM, 99.9%), acetone (99.8%, ExtraDry), and pentane (99.5%) were purchased from Fischer Scientific. Sodium tetrakis[3,5-bis(trifluoromethyl)phenyl]-borate ($NaBAr^F_4$, 97%) was purchased from Matrix Scientific and used as received. Chloromethyl(1,5-cyclooctadiene) palladium(II) (99%) was purchased from Stream Chemicals, Inc. and used as received. Solvents involved in handling catalysts and polymers are obtained from the solvent purification system (Inert PureSolv MD 5) or dried over molecular sieves (4 Å).

1.2 Materials: Block Copolymer Synthesis

Monomers: methyl acrylate from Sigma-Aldrich (99%). Monomer was purified via passing through a basic alumina column.

Initiators: 2-Hydroxyethyl α-bromoisobutyrate was obtained from TCI Chemicals (> 97.0%). Initiators were used as received.

Macroinitiators: α-Bromoisobutyrate terminated polyethylene-based macroinitiators with different molecular weights were obtained from the University of Houston. The macroinitiators were stored in the freezer.

Catalyst Complex: Copper(II) bromide ($CuBr_2$) was obtained from Sigma-Aldrich (99%). Copper(0) wire obtained from Arcor electronics (99.9% pure, 0.51 mm diameter). Copper(0) wire was reduced using a mixture of MeOH and HCl in a 1:1 ratio with a stir bar. Me_6Tren (tris[2-(dimethylamino)ethyl]amine) was received from Ambeed (97%). Copper(II) bromide and Me_6Tren were used as received.

Solvents: Chlorobenzene obtained from TCI Chemicals (> 98%). Chloroform-D "100%" obtained from Cambridge Isotope Laboratories (D, 99.96%). DMF obtained from Thermo Scientific Chemicals (99.8+%, ACS). All solvents used as received.

1.3 Instrumentation (Macroinitiator Synthesis)

Nuclear Magnetic Resonance (NMR) – ^{1}H and ^{13}C NMR spectra were acquired at room temperature on a JOEL JNM-ECA 400 (400 MHz), JNM-ECZ400S (400 MHz), JNM-ECZ500R (500 MHz), or ECA-600 (600 MHz). Chemical shifts were measured relative to residual solvent peaks as an internal standard set to δ 5.32 and δ 54.00 (methylene chloride-d_2, (CD_2Cl_2)) and δ 7.26 and δ 77.16 (chloroform-d ($CDCl_3$)) and δ 6.00 (1,1,2,2-tetrachloroethane-d2 (TCE-d_2)). Diffusion-ordered NMR spectroscopy (DOSY) of block copolymers was performed on the ECA-600. The parameters of the experiments for the diffusion time were set to 0.4 s, delta = 4 ms, and relaxation delay = 7 s. An exponential array function was applied between 3 mT/m and 280 mT/m for points =16 with 8 scans.

Gel Permeation Chromatography (GPC) – Polymer samples for polyethylene and block copolymers were analyzed using a Tosoh high-performance GPC system HLC-8320 equipped with an auto-injector, a built-in dual differential refractive index (RI) detector, and TSKgel G series columns connected in series (7.8 x 300 mm TSKgel G5000Hxl, TSKgel G4000Hxl, TSKgel G3000Hxl). The GPC analyses were carried out in HPLC grade tetrahydrofuran (THF) with a flow rate of 1 ml min^{-1} at 40 °C. GPC analysis weights (M_n and M_w) and dispersity (*Đ*) were calculated from polystyrene (PS) standards with a molar mass of 800 to 2.2×10^6 g mol^{-1} provided by Polymer Standard Service (PSS).

Elemental Analysis – Analyses were performed by Atlantic Microlabs Inc. CHNS analyses were performed by combustion using automatic analyzers. For compounds containing bromine, analyses were performed by flask combustion followed by ion chromatography. All analyses are measured by weight determination.

1.4 Instrumentation (Block Copolymer Synthesis)

In order to confirm the conversion of the monomer and the chain end integrity, ^{1}H nuclear magnetic resonance (^{1}H NMR) was used. The ^{1}H NMR machine was Bruker Avance TM III 500 MHZ spectrometer. In order to confirm the molecular weight and dispersity of the homopolymers and block copolymers, gel permeation chromatography (GPC) was used. The GPC used were as follows: Waters 515 pump and a Water 2414 differential refractometer were used with PSS columns (SDV 10^5, 10^3, and 500 Å). Measurement condition for GPC is as follows: 35 °C with the flow rate of 1 ml min^{-1}. Tetrahydrofuran as eluent and linear poly(methyl methacrylate) standard used for calibrations.

2. Procedures for Macroinitiator Synthesis

2.1 Ligands and Catalysts

A) Synthesis of bis(2,6-diisopropylaniline)-butane-2,3-diimine

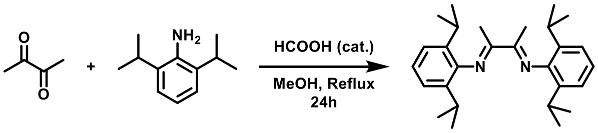

B) Synthesis of pre-catalyst 1A and catalyst Pd-HFIP

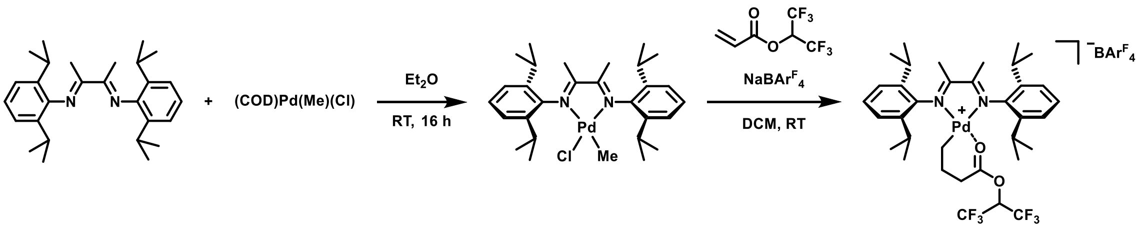

C) Synthesis of 2-hydroxyethyl α-bromoisobutyrate (HOBIB)

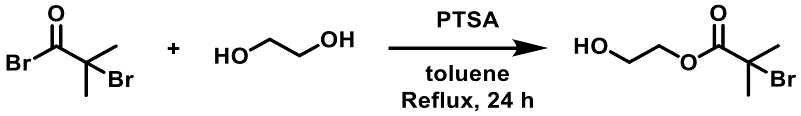

D) Synthesis of 2-bromo-N-(2-hydroxyethyl)-2-methylpropionamide (HOBIBA, 2-hydroxyethyl α-bromoisobutyramide)

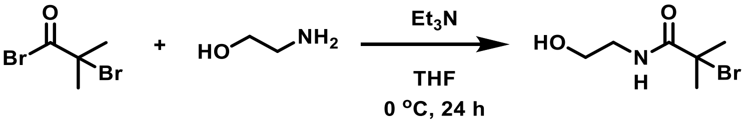

Figure S1. (A) Synthetic scheme of bis(2,6-diisopropylaniline)-butane-2,3-diimine (B) Synthetic scheme of (Ar-N=C(Me)-C(Me)=N-Ar)Pd(Me)(Cl) (Ar = 2,6-diisopropylaniline) (Pre-Catalyst 1A) and $[(Ar\text{-}N{=}C(Me)\text{-}C(Me){=}N\text{-}Ar)Pd(CH_2)_3C(O)OCH(CF_3)_2]^+[B(3,5\text{-}C_6H_3(CF_3)_2)_4)]^-$

(Catalyst Pd-HFIP) (C) Synthetic scheme of 2-hydroxyethyl 2-bromo-2-methylpropanoate (HOBIB) (D) Synthetic scheme of 2-bromo-N-(2-hydroxyethyl)-2-methylpropionamide (HOBIBA)

Bis(2,6-diisopropylaniline)-butane-2,3-diimine was prepared according to literature precedents.[1, 2] The compound is known. ^{1}H NMR (400 MHz, CD_2Cl_2, ppm): δ 7.18 (m, 4H), 7.10 (m, 2H), 2.73 (s, J = 6.60 Hz, 4H), 2.07 (s, 6H), 1.22 (d, J = 5.93 Hz, 6H), 1.20 (d, J = 5.93 Hz, 6H), 1.18 (d, J = 5.93 Hz, 6H), 1.16 (d, J = 5.93, 6H).

Pre-catalyst 1A was synthesized using a modified literature procedure.[1, 2] This compound is known. ^{1}H NMR (400 MHz, $CDCl_3$, ppm): δ 7.36 – 7.26 (m, 6H), 3.08 (sep, J = 6.60 Hz, 2H), 3.02 (sep, J = 6.93 Hz, 2H), 2.06 (s, 3H), 2.05 (s, 3H), 1.42 (d, J = 7.31 Hz, 6H), 1.37 (d, J = 7.31 Hz, 6H), 1.20 (d, J = 6.99 Hz, 6H), 1.19 (d, J = 6.99 Hz, 6H), 0.39 (s, 3H).

2-Hydroxyethyl 2-bromo-2-methylpropanoate (HOBIB) was synthesized using a modified literature procedure. This is a known compound. ^{1}H NMR (400 MHz, $CDCl_3$, ppm): δ 1.96 (s, 6H), 3.87 (t, J = 4.7 Hz, 2H), 4.31 (t, J = 4.7 Hz, 2H).

2-Bromo-*N*-(2-hydroxyethyl)-2-methylpropionamide (HOBIBA) was synthesized using a modified literature procedure. This is a known compound. ^{1}H NMR (400 MHz, $CDCl_3$, ppm): δ 7.13 (br, 1H), 3.72 (m, 2H), 3.42 (m, 2H), 2.32 (br, 1H), 1.93 (s, 6H)

Synthesis of [(Ar-N=C(Me)-C(Me)=N-Ar)Pd$(CH_2)_3$C(O)OCH$(CF_3)_2$]$^+$(BAr$^F{}_4$)$^-$

Pd-HFIP

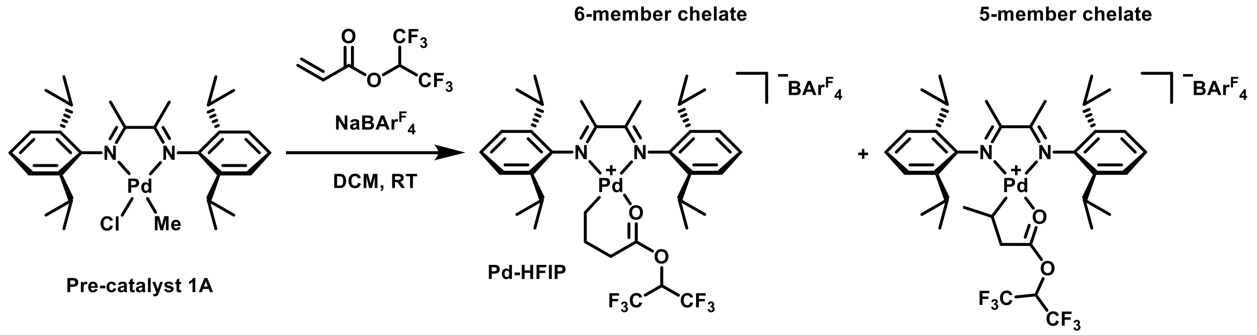

Figure S2. Synthesis of Pd-HFIP complex

At room temperature, palladium pre-catalyst 1A (500 mg, 0.882 mmol, 1 eq) was weighted out with NaBAr$^F{}_4$ (845 mg, 0.978 mmol, 1.1 eq) in a flame-dried round bottom flask. DCM (20 ml) and 1,1,1,3,3,3-hexafluoroisopropyl acrylate (244.3 mg, 1.1 mmol, 1.2 eq) were mixed and then injected into the reaction. After stirring for 1 hour, NaCl was removed from the reaction via filtration using a 0.45 µm PTFE syringe filter. The solvent was then evaporated, and the residue was crashed out and washed with pentanes (100 ml). The complex was collected through vacuum filtration and dried under a high vacuum overnight. Catalyst Pd-HFIP ester was obtained as an orange solid (yield = 91%). Note that the catalyst is susceptible to hydrolysis. Crystals for X-Ray characterization were obtained by layering pentanes over a solution of the complex in DCM at -20 °C.

^{1}H NMR (600 MHz, CD$_2$Cl$_2$) δ 7.68 (br, 8H, BArF, H_o), 7.51 (br, 4H, BArF, H_p), 7.40 – 7.29 ($6H_{aryl}$), 4.10 – 4.07 (m, J = 4.08 Hz, 1H CH(CF$_3$)$_2$), 2.87 – 2.83 (m, J = 2.85, 2H, CH(CH$_3$)$_2$), 2.81 – 2.76 (m J = 2.79 Hz, 2H, CH(CH$_3$)$_2$), 2.63 – 2.61 (t, J = 2.62 Hz, 2H, CH$_2$CH$_2$CH$_2$C(O)), 2.19 (s, J = 2.20 Hz, 3H, N=C(CH$_3$)-C'(CH$_3$)=N), 2.17 (s, J = 2.17 Hz, 3H, N=C(CH$_3$)-C'(CH$_3$)=N), 1.41 – 1.39 (t, J =1.40 Hz, 2H, CH$_2$CH$_2$CH$_2$C(O)), 1.32 – 1.31 (d, J = 1.31 Hz, 6H, CH(CH$_3$)$_2$), 1.25 – 1.24 (d, J = 1.24 Hz, 6H, CH(CH$_3$)$_2$), 1.21 – 1.19 (d, J = 1.20 Hz, 6H, CH(CH$_3$)$_2$), 1.16 – 1.15 (d, J = 1.16 Hz, 6H, CH(CH$_3$)$_2$), 0.71 – 0.68 (p, J = 0.69 Hz, 1H, PdCH$_2$CH$_2$CH$_2$C(O)).

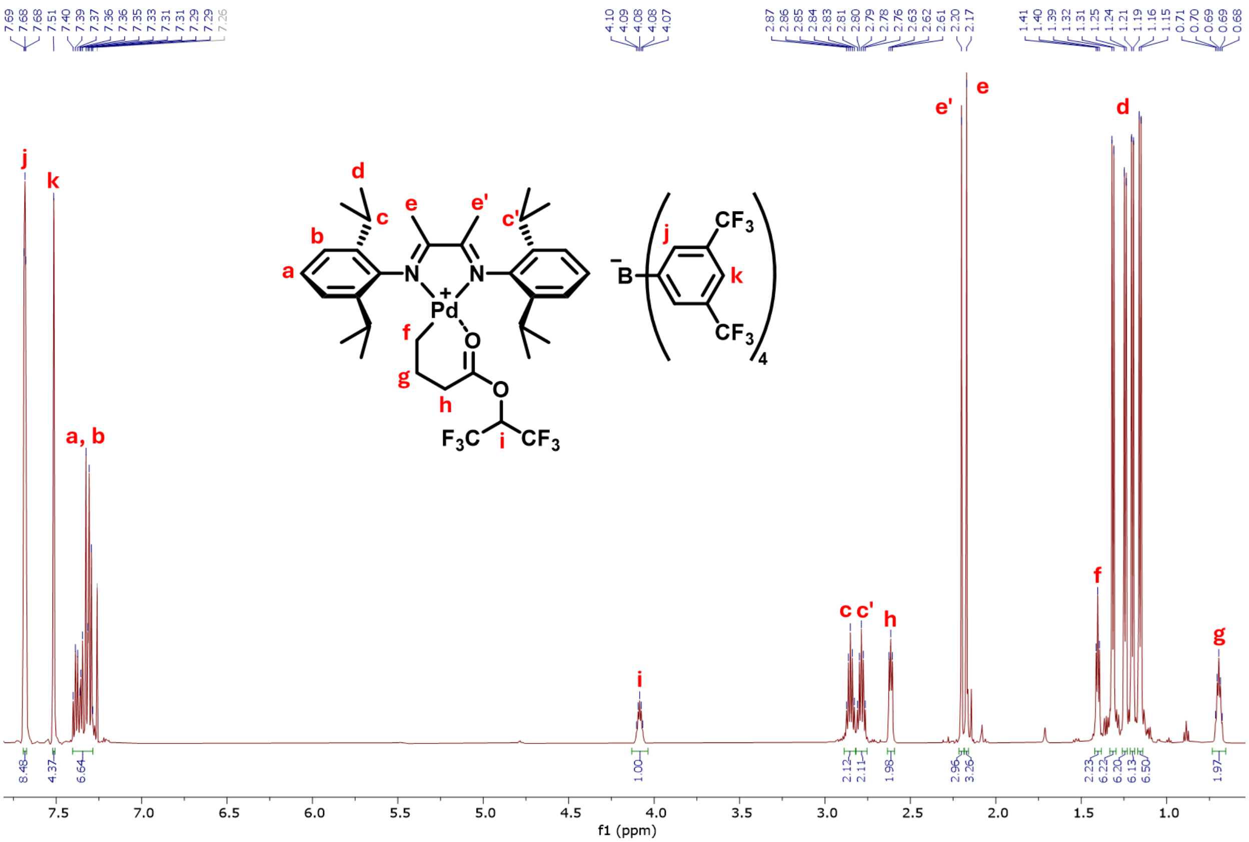

Figure S3. ^{1}H NMR of Pd-HFIP complex

^{13}C NMR (600 MHz, CD_2Cl_2) δ 181.70 (C=O), 178.94 and 171.30 (C=N), 162.23, 161.90, 161.57, 161.24 (C_{ipso} – $BAr^F{}_4$), 140.01 and 139.73 (C_{ipso} – Aryl), 138.04 and 137.36 (C_{ortho} – Aryl), 134.87 (C_{ortho} – $BAr^F{}_4$), 129.03 and 128.83 (C_{para} – Aryl), 127.32 (C_{meta} – $BAr^F{}_4$), 125.52, 124.82, 124.40, 123.71 (C_{meta} – Aryl), 121.90 (CF_3 – $BAr^F{}_4$), 119.94 and 118.06 ($CH(CF_3)_2$), 117.55 (C_{para} – $BAr^F{}_4$), 35.08 ($CH_2CH_2CH_2C(O)$), 30.52 ($PdCH_2CH_2CH_2C(O)$), 29.22 and 28.97 ($CH(CH_3)_2$), 23.34 ($CH_2CH_2CH_2C(O)$), 22.92 ($CH(CF_3)_2$), 21.14 and 19.56 ($N{=}C(CH_3)$).

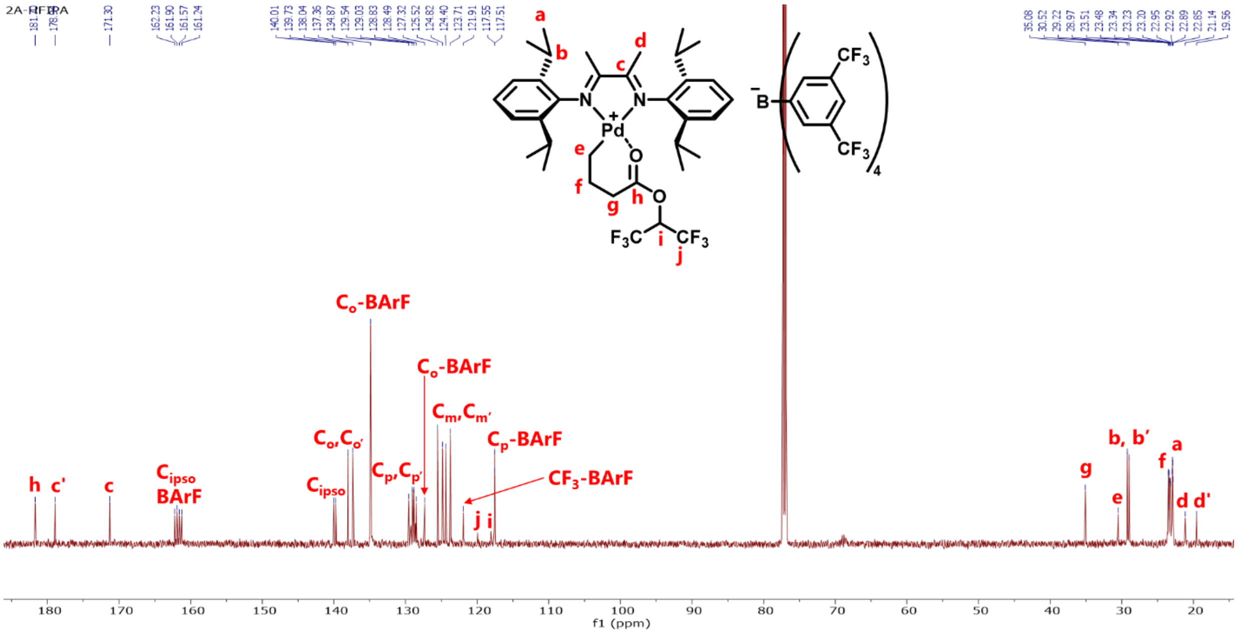

Figure S4. ^{13}C NMR of Pd-HFIP complex

^{19}F NMR (400 MHz, CD_2Cl_2) δ -62.31 (s, 6F, $CH(CF_3)_2$, 6-member chelate), -72.66 (s, 6F, $CH(CF_3)_2$, 5-member chelate)

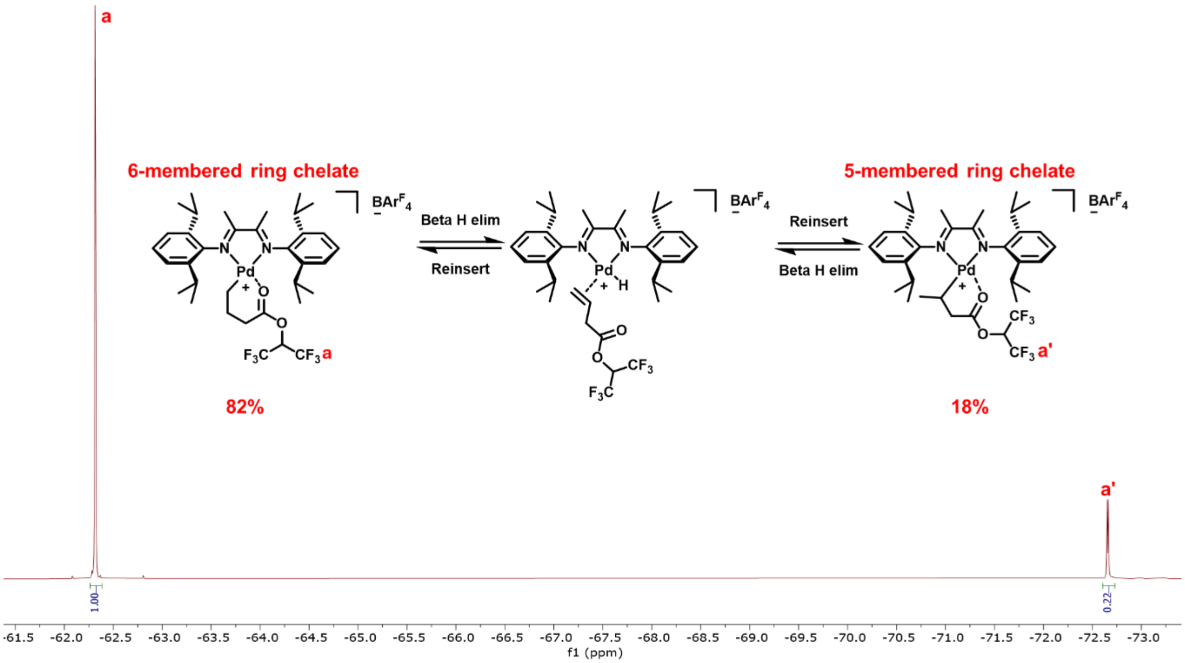

Figure S5. ^{19}F NMR of Pd-HFIP complex

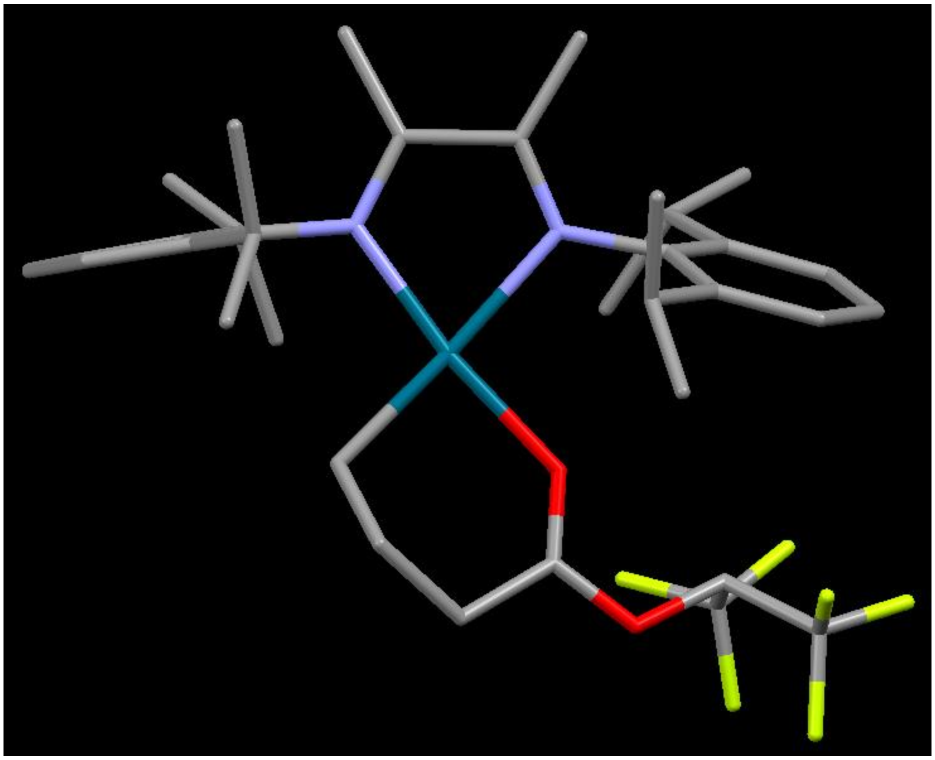

Figure S6. X-Ray diffraction analysis of catalyst Pd-HFIP

2.2 Macroinitiator Synthesis

Ethylene polymerization using Pd-HFIP complex

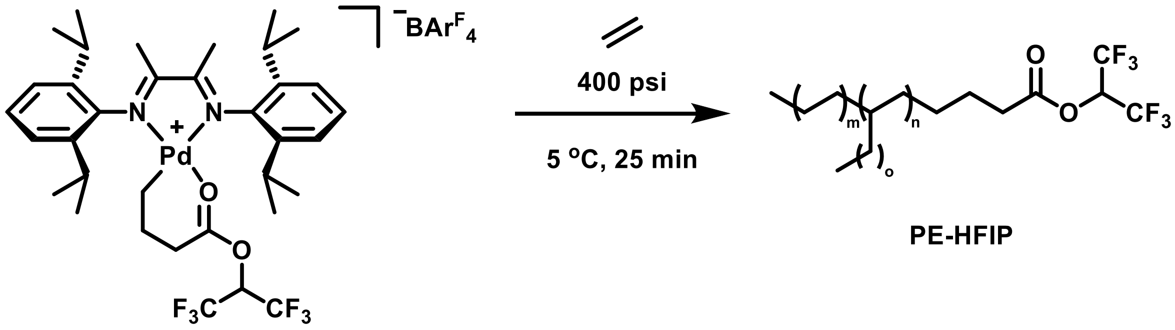

Figure S7. Synthesis of highly branched polyethylene chain with hexafluoroisopropyl functional group (PE-HFIP), using catalyst Pd-HFIP.

A mechanically stirred 300 mL Parr® reactor equipped with a heating mantle and a thermocouple was heated overnight to 80 °C under vacuum. The reactor was vented with argon and then cooled to room temperature. The reactor was then pressurized to 100 psi ethylene and vented three times. A sampling port located on the reactor was opened and chlorobenzene (80 ml) was added to the reactor. After closing the sampling port, the reactor was pressurized with ethylene to 100 psi and vented twice. The ethylene pressure was then raised to 400 psi, and the chlorobenzene was stirred until the system equilibrated at 5 °C After venting the ethylene pressure, a solution of catalyst Pd-HFIP (100 mg, 6.2×10^{-5} mol) in chlorobenzene (20 ml) was added to the reactor through the sampling port. The reactor was then recharged with ethylene to 400 psi to start the polymerization. After the allotted polymerization time (25 min), the ethylene pressure was vented and 500 µL of triethylsilane was added to the reaction to quench the catalyst. The reaction

solution was then allowed to stir overnight resulting in the formation of palladium black, which was removed by gravity filtration. The reaction was transferred to a round bottom flask from which the solvent was removed under reduced pressure. The residue was dissolved in hexanes then precipitated in dry acetone and centrifuged at 7800 rpm for thirty minutes (repeated three times). The polymer was then dissolved in hexanes and passed through a silica plug via vacuum filtration. The isolated polymer was dried before analysis.

For the kinetic study, the evolution of molecular weight over time was investigated for ethylene homopolymerization under the same reaction condition discussed in the above paragraph. A small amount (~0.5 mL) of aliquots were removed from the Parr® reactor at different time intervals (15, 30, 45, 60, 90, 120, and 150 minutes) while maintaining the continuous flow of ethylene and collected in 1-dram vials containing 0.1 mL of triethylsilane to quench the reaction. The mixture was evaporated and dried under vacuum. The residue was then dissolved in THF, filtered, and analyzed by GPC.

Table S1. Kinetic data of insertion polymerization of ethylene

Entry	Ethylene (psi)	PhCl (mL)	Pd-HFIP (mg)	Temp (°C)	Time (min)	M_n[a] (kg/mol)	*Đ*[a] (M_w/M_n)
1					15	10.0	1.02
2					30	17.2	1.01
3					45	24.5	1.01
4	400	100	1000	5	60	30.9	1.01
5					90	43.5	1.01
6					120	55.5	1.02
7					150	68.1	1.01

[a]Molar mass (M_n) and dispersity (*Đ*) were determined by gel permeation chromatography (GPC) analysis with samples ran in THF at 40 °C calibrated to polystyrene standards.

General procedure for transesterification of hexafluoroisopropyl ester functionalized polyethylene (PE-HFIP):

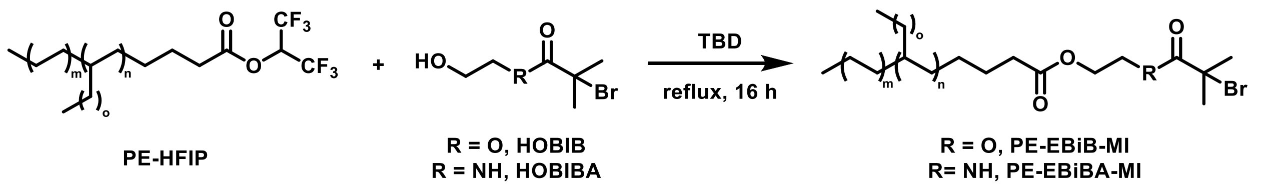

Figure S8. Synthesis of PE-EBiB-MI, polyethylene-based macroinitiator terminated with ethyl bromoisobutyrate, and PE-EBiBA-MI, polyethylene-based macroinitiator terminated with ethyl bromoisobutyramide.

In an oven-dried round bottom flask equipped with a magnetic stir bar, PE-HFIP (1 eq) and 1,5,7-triazabicyclo[4.4.0]dec-5-ene (TBD, 0.5 eq) were dissolved in toluene, injected and allowed to stir at 45 °C under flow of N_2 for 20 minutes. It is necessary to use a catalytic amount of TBD to avoid the side reaction and the formation of homo-coupled byproduct or elimination byproduct. The temperature was then raised to 85 °C followed by injection of the alcoholic nucleophile (HOBIB or HOBIBA, 1.2 eq) pre-mixed in toluene (0.0052 M). Only a small excess of alcohol is necessary to simplify the purification process. The reaction was then purged with N_2, sealed with a septum, and allowed to stir at 85 ºC overnight under flow of N_2 to expel the generated hexafluoroisopropanol. The reaction vessel was then allowed to cool to room temperature before the polymer solution was concentrated under reduced pressure. The concentrated polymer was dissolved in a minimal amount of hexanes and crashed into acetone before being centrifuged at 7800 rpm for thirty minutes (repeated three times) to remove any excess nucleophile (HOBIB or HOBIBA). The polymer was then passed through a plug of neutral alumina stacked on top of silica

by gravity to remove the triazabicyclo[4.4.0]dec-5-ene (TBD). After the column, a small aliquot was taken, dried, and then examined by proton NMR to ensure purity. It was necessary to repeat the crashing, centrifugation, and column steps if any excess alcohol was present in the proton NMR to obtain a high purity macroinitiator. The solvent was removed under reduced pressure and then the obtained polymer was dried under a high vacuum at 40°C overnight.

3. Ester-linked tertiary-bromide capped initiators

3.1 PMA Homopolymer synthesis via SARA ATRP

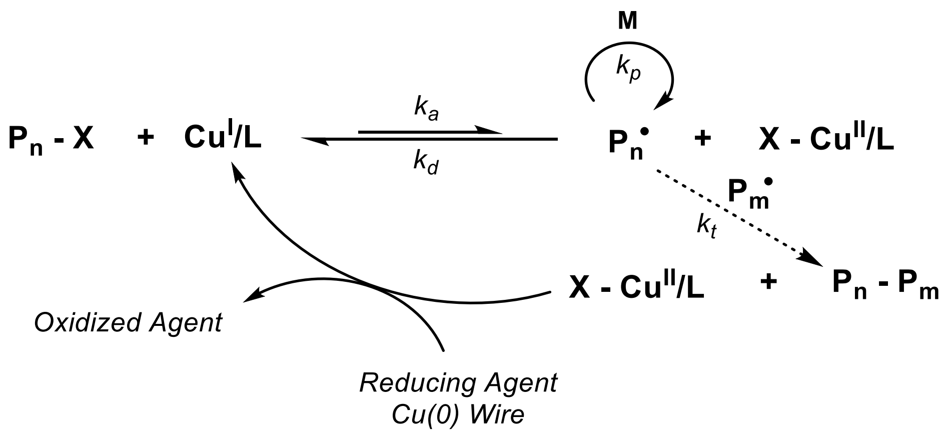

Figure S9. Top) General scheme of SARA ATRP, Bottom) The synthetic scheme of PMA via SARA ATRP.

Figure S9 shows the general scheme of the SARA ATRP. To synthesize poly(methyl acrylate) homopolymer: the pressure-release vial was vacuumed and deoxygenated via backfilling with N_2 three times. Monomer and solvents (methyl acrylate and chlorobenzene) were deoxygenated with N_2 for 30 minutes. Chlorobenzene and monomer, specified amount for each experiment in the caption of the figures and table, were added to the reaction vial along with the catalyst complex ($CuBr_2$ and Me_6Tren) solution and the initiator solution (2-hydroxyethyl α-bromoisobutyrate). Copper wire reduced with methanol and hydrochloric acid was added to the reaction vial, based on the experimental condition specified in the caption of the figures / tables. The reaction vial was further degassed with N_2 for 10 minutes.

3.2 PE-*b*-PMA Block Copolymer Synthesis via SARA ATRP

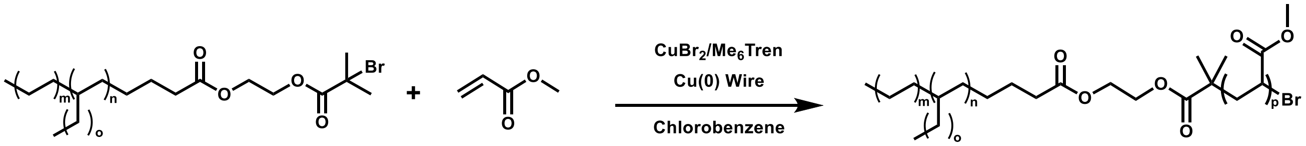

Figure S10. Synthetic scheme of PE-*b*-PMA via SARA ATRP, using PACE-prepared highly branched polyethylene macroinitiator PE-EBiB-MI.

Figure S10 shows the synthetic procedure of the polyethylene-*block*-polyacrylate block copolymer. In the pressure release vial, appropriate amount of macroinitiator (specified in the experimental procedure / caption of each figure) was added. The pressure-release vial was vacuumed and deoxygenated via backfilling with N_2 three times. Chlorobenzene and monomer (acrylate species) were deoxygenated with N_2 for 30 minutes. Appropriate amount of solvent and monomer (specified amount for the experiment in the caption) were added to the pressure-release vial using syringe to maintain oxygen-free condition. Catalyst-complex solution was added to the reaction vial. The specified length of copper wire was added to the reaction vial and further degassed with nitrogen for 10 minutes.

3.3 Block copolymer PE-*b*-PMA 2D NMR Results (Diffusion-ordered spectroscopy, DOSY)

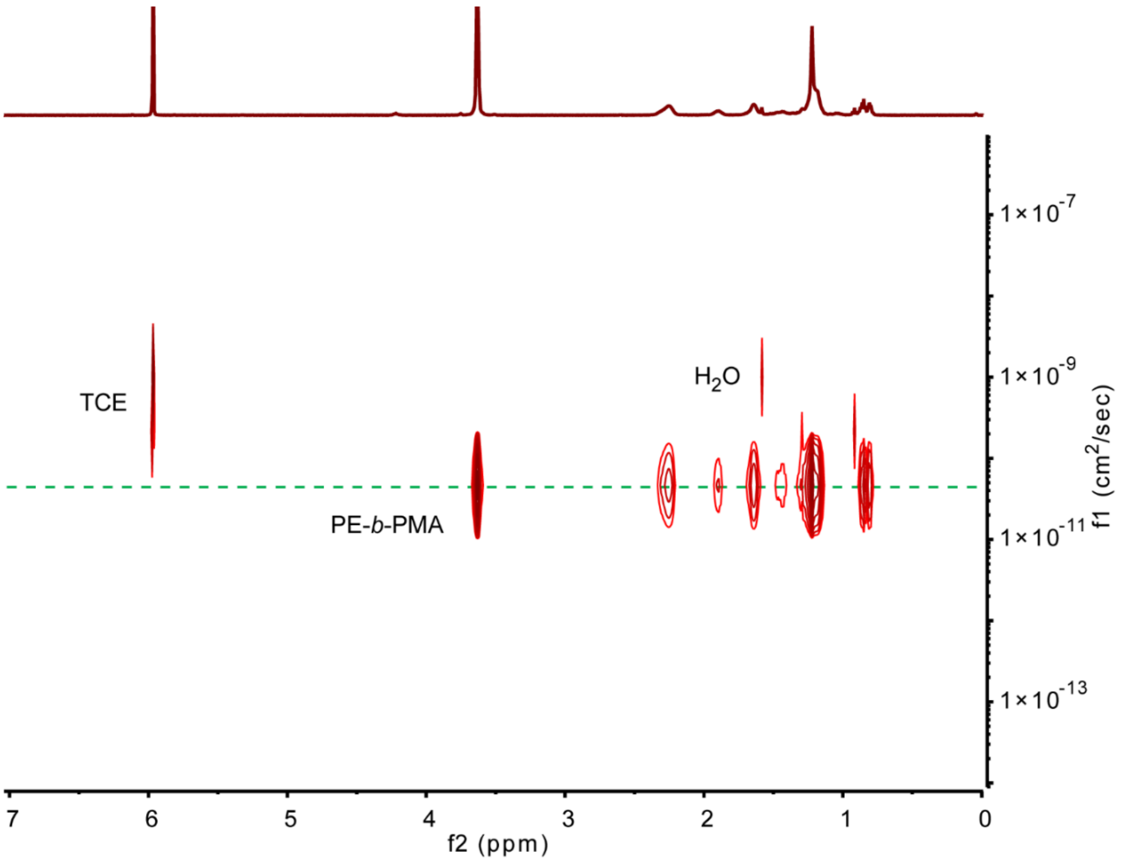

Figure S11. DOSY ^{1}H NMR spectrum of purified PE-*b*-PMA in TCE ($C_2D_2Cl_4$) at 25 °C.

Figure S11 showed purified PE-*b*-PMA block copolymer analyzed via ^{1}H DOSY NMR. The obtained signals indicated that to polyethylene block (1.25-0.83) ppm and poly(methyl acrylate) block (3.66, 2.32, 1.97, 1.68) were aligned in a single diffusion coefficient. This observation confirmed that formed blocks were covalently bonded with each other.

4. Calculation method for block copolymer initiation efficiency

Block copolymer initiation efficiency is calculated from the deconvolution of the GPC traces. First, the original GPC traces (**Figure S12**, Blue Dash) were converted to chain-number distribution. (**Figure S12**, Black) The refractive index at each point of the elution volume was divided by the calibrated molecular weight. Following the conversion from weight-based traces to number-based chromatogram, the bimodal peak was deconvoluted to fit the BCP peak (low elution volume) and unfunctionalized PE and uninitiated macroinitiators peak (high elution volume) using OriginLabs software. In the example below, the PACE-prepared polyethylene-based amide-linked macroinitiator was used for the synthesis of PE-*b*-PMA block copolymer. In this example, a macroinitiator with a MW of 12,000 is used.

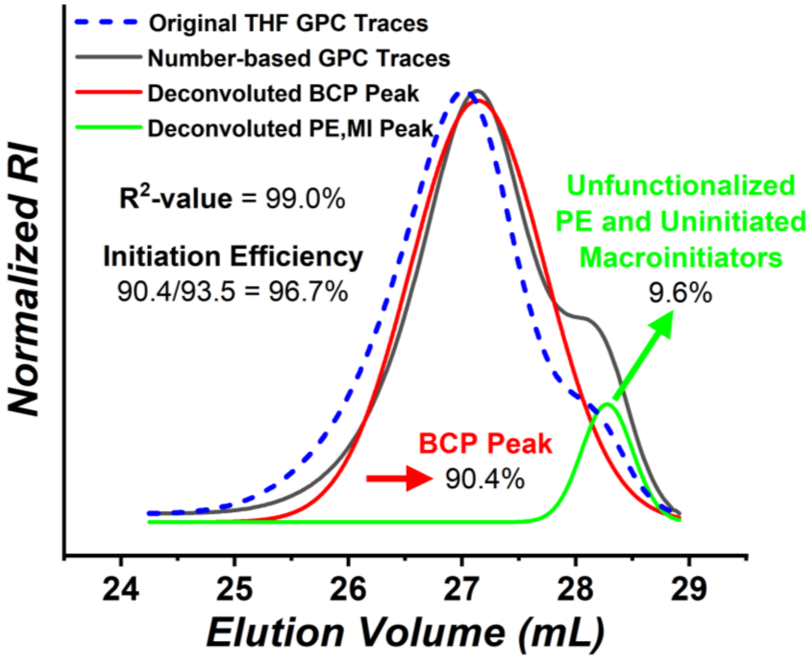

Figure S12. GPC chromatogram of the block copolymer analyzed via deconvolution. (Blue, short dash) Original THF GPC traces, based on polymer weights (Black, solid) chain-number based distribution (Red, solid) block copolymer peak (Green, solid) peak assigned to the unfunctionalized polyethylene and uninitiated macroinitiators. Amide-linked macroinitiator PE-

EBiBA-MI: Mn = 12,000, *Đ* = 1.01. Synthesis condition: [Macroinitiator]:[MA]:[$CuBr_2$]:[Me_6Tren] = 1:500:0.04:0.1 $[MA]_0$ = 5.05 M. Total reaction volume = 0.88 ml. Chlorobenzene (45 vol%) and DMF (9 vol%) used as solvent, reaction time = 6.5 h, Cu^0 wire surface area = 1.57 cm^2. All reagents deoxygenated with N_2 prior to block copolymerization.

5. Amide-linked tertiary-bromide capped initiators

5.1 Amide-linked PACE-prepared MI synthesis: PE-EBiBA-MI

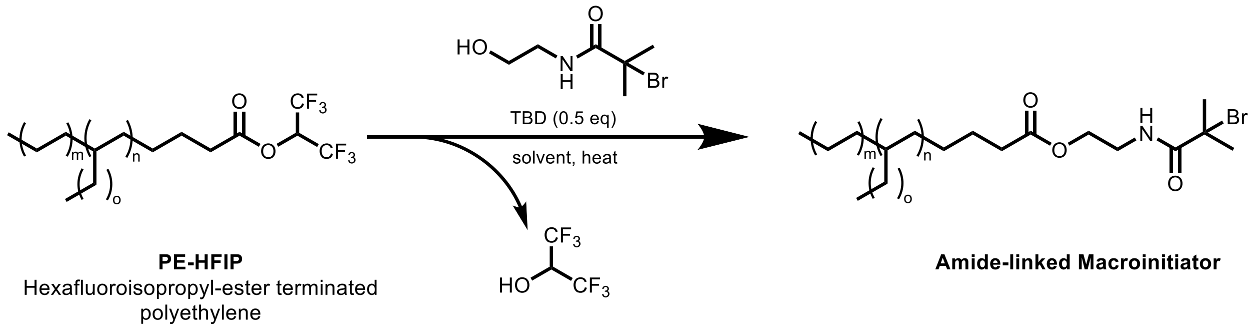

Figure S13. The last step of the amide-linked polyolefin macroinitiator synthesis scheme, yielding the macroinitiator via Polyolefin Active Ester exchange (PACE) approach.

5.2 Elimination side reaction for amide linked MI, PE-EBiBA-MI

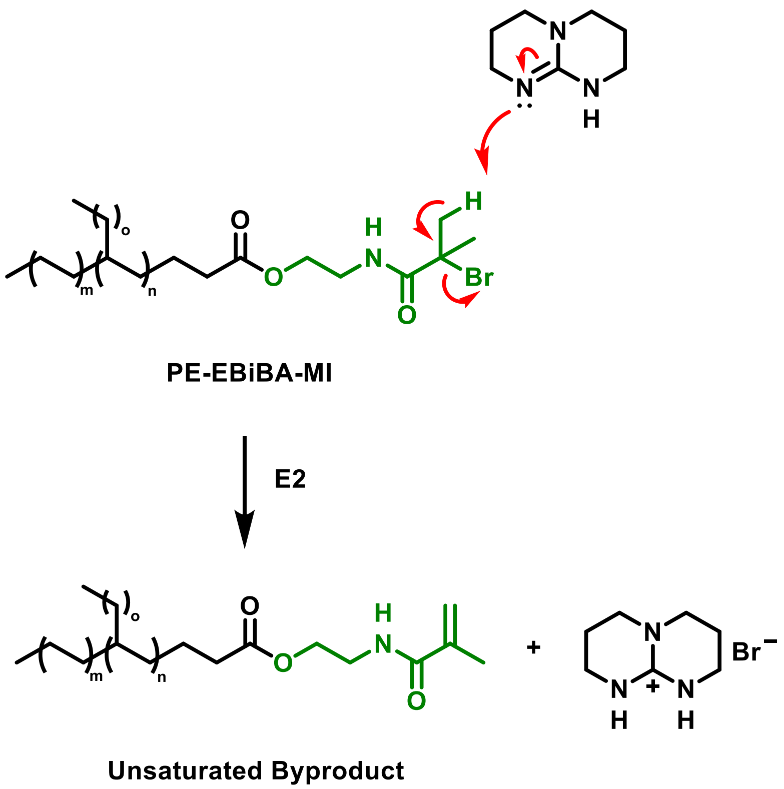

Figure S14. Proposed mechanism for the formation of the unsaturated byproduct during the synthesis of PE-EBiBA-MI

When synthesizing the PE-EBiBA-MI, we observe a small amount of unsaturated product. This product could form through an E2 mechanism (**Figure S14**) due to a strong base (TBD) and a good leaving group (bromide).

References

1. Adelsberger, J.; Kulkarni, A.; Jain, A.; Wang, W.; Bivigou-Koumba, A. M.; Busch, P.; Pipich, V.; Holderer, O.; Hellweg, T.; Laschewsky, A.; Müller-Buschbaum, P.; Papadakis, C. M., Thermoresponsive PS-b-PNIPAM-b-PS Micelles: Aggregation Behavior, Segmental Dynamics, and Thermal Response. *Macromolecules.* **2010,** *43* (5), 2490-2501.

2. Convertine, A. J.; Lokitz, B. S.; Vasileva, Y.; Myrick, L. J.; Scales, C. W.; Lowe, A. B.; McCormick, C. L., Direct Synthesis of Thermally Responsive DMA/NIPAM Diblock and DMA/NIPAM/DMA Triblock Copolymers via Aqueous, Room Temperature RAFT Polymerization. *Macromolecules.* **2006,** *39* (5), 1724-1730.
