# Supplementary material for: Polyethylene-poly(methyl acrylate) Block Copolymers from PACE-SARA ATRP: Utilizing Polyolefin Active Ester Exchange-Based Macroinitiators in Atom Transfer Radical Polymerization
Source: Macromolecules. 2025 Jan 30;58(3):1337–48. doi: 10.1021/acs.macromol.4c02684 (PMC11823591; doi:10.1021/acs.macromol.4c02684)
Supplement: Supplementary file 3 — ma4c02684_si_003.pdf [file ma4c02684_si_003.pdf]

2A-HFIPA_XW626

Table 1 Crystal data and structure refinement for 2A-HFIPA_XW626.

Identification code	2A-HFIPA_XW626
Empirical formula	$C_{67}H_{59}BF_{30}N_2O_2Pd$
Formula weight	1611.37
Temperature/K	123(2)
Crystal system	orthorhombic
Space group	$P2_12_12$
a/Å	16.2624(6)
b/Å	35.9505(14)
c/Å	11.9895(5)
α/°	90
β/°	90
γ/°	90
Volume/Å^3	7009.6(5)
Z	4
ρ_{calc}g/cm^3	1.527
μ/mm^{-1}	0.389
F(000)	3248.0
Crystal size/mm^3	0.48 × 0.35 × 0.22
Radiation	MoKα (λ = 0.71073)
2Θ range for data collection/°	3.378 to 61.088
Index ranges	$-23 \le h \le 23$, $-50 \le k \le 51$, $-15 \le l \le 17$
Reflections collected	140414
Independent reflections	21345 [R_{int} = 0.0197, R_{sigma} = 0.0135]
Data/restraints/parameters	21345/726/1038
Goodness-of-fit on F^2	1.029
Final R indexes [I>=2σ (I)]	R_1 = 0.0246, wR_2 = 0.0644
Final R indexes [all data]	R_1 = 0.0263, wR_2 = 0.0652
Largest diff. peak/hole / e Å^{-3}	0.55/-0.42
Flack parameter	-0.0263(18)

Table 2 Fractional Atomic Coordinates (×10^4) and Equivalent Isotropic Displacement Parameters (Å^2×10^3) for 2A-HFIPA_XW626. U_{eq} is defined as 1/3 of the trace of the orthogonalised U_{IJ} tensor.

Atom	*x*	*y*	*z*	U(eq)
Pd1	8133.1(2)	3614.2(2)	5493.0(2)	21.83(3)
F1	5547.5(12)	2148.5(5)	5504(2)	78.0(7)
F2	7661.1(11)	2504.3(6)	7050.1(18)	63.5(5)
F3	5126.1(11)	2710.8(6)	5352(2)	75.2(6)
F4	5118.3(13)	2446.5(6)	6947(2)	81.5(7)
F5	6593.4(15)	2474.5(6)	8099.8(18)	73.2(6)
F6	6836.8(14)	2044.2(4)	6898.2(18)	65.9(5)
F9	5097.0(10)	4566.1(5)	-2508.4(11)	46.5(4)
F15	2275.7(9)	3196.9(4)	5551.1(16)	50.0(4)
F16	3143.6(13)	4938.6(4)	4166.3(12)	50.6(4)
F18	-329.9(11)	3542.1(7)	531(3)	98.0(9)
F19	3911.3(10)	4773.5(4)	-2008.8(12)	43.2(3)
F20	4046.9(10)	4204.3(4)	-2504.8(11)	41.5(3)
F23	3201.1(15)	3401.3(5)	6633.2(13)	65.1(5)
F24	3514.2(13)	3030.1(5)	5321.9(19)	74.9(7)
F25	2702.3(12)	4731.5(5)	5723.9(16)	61.3(5)
F26	3987.0(9)	4770.1(4)	5443.6(15)	45.8(3)
F29	212.1(14)	3351.4(7)	2024.7(19)	79.4(7)
F30	600.7(13)	3122.5(6)	510(3)	90.8(8)
O1	7381.7(8)	3154.2(3)	5557.1(14)	28.6(3)
O2	6809.5(10)	2610.5(4)	5172.6(14)	35.2(3)
N1	8805.8(9)	4083.3(4)	5664.4(14)	24.2(3)
N2	7455.3(9)	3910.6(4)	6725.3(13)	20.8(3)
C1	9572.3(11)	4134.5(5)	5079.2(17)	26.5(4)
C2	9540.2(13)	4265.4(6)	3978(2)	32.8(4)
C3	10282.1(14)	4281.8(8)	3392(2)	40.0(5)
C4	11009.5(14)	4167.5(8)	3866(2)	42.7(6)
C5	11017.3(14)	4040.9(8)	4956(2)	42.3(5)
C6	10305.6(12)	4022.9(6)	5595(2)	33.4(4)
C7	7738.0(11)	4236.8(5)	6944.0(16)	22.9(3)
C8	8536.2(11)	4327.8(5)	6369.5(17)	24.4(3)
C9	8966.8(13)	4684.7(6)	6637(2)	33.8(4)
C10	8744.3(14)	4389.2(9)	3427(2)	44.3(6)
C11	8637(2)	4229.5(10)	2257(3)	56.2(7)
C12	10332.4(15)	3876.0(8)	6785(2)	41.8(5)
C13	11131(2)	3983.8(11)	7386(3)	61.4(8)
C14	8770.4(14)	3372.8(6)	4237(2)	35.5(5)
C15	8184.0(17)	3189.5(6)	3420.2(19)	39.0(5)

Table 2 Fractional Atomic Coordinates (×10[4]) and Equivalent Isotropic Displacement Parameters (Å[2]×10[3]) for 2A-HFIPA_XW626. U_{eq} is defined as 1/3 of the trace of the orthogonalised U_{IJ} tensor.

Atom	*x*	*y*	*z*	U(eq)
C16	7780.7(15)	2836.7(6)	3856(2)	37.2(5)
C17	7323.5(12)	2890.6(5)	4922.6(18)	28.1(4)
C18	6698.2(10)	3767.0(5)	7161.4(15)	21.8(3)
C19	6672.3(12)	3627.3(6)	8257.2(16)	28.4(3)
C20	5934.7(15)	3464.2(7)	8603(2)	37.3(5)
C21	5268.2(14)	3437.1(7)	7889(2)	38.8(5)
C22	5319.0(12)	3568.6(6)	6813(2)	32.4(4)
C23	6037.9(11)	3735.0(5)	6416.5(16)	23.2(3)
C24	7337.5(13)	4517.1(6)	7685.4(18)	30.5(4)
C25	8706(2)	4815.8(10)	3394(3)	63.8(9)
C26	10223(2)	3458.3(10)	6823(3)	56.2(7)
C27	6388.3(14)	2630.5(6)	6217(2)	37.5(5)
C28	5536.3(17)	2479.4(8)	6004(3)	56.6(8)
C29	7434.3(15)	3628.6(8)	8996.6(18)	38.4(4)
C30	8015.7(18)	3313.7(9)	8655(2)	52.2(7)
C31	6099.1(11)	3881.8(5)	5228.8(16)	25.2(3)
C32	5835(2)	4288.3(7)	5167(2)	45.4(6)
C33	6872(2)	2409.3(7)	7081(3)	49.3(6)
C34	7239(2)	3597.4(11)	10240(2)	59.7(7)
C35	5619.1(16)	3653.2(7)	4383(2)	43.2(5)
C36	3709.2(12)	3386.2(5)	1202.1(16)	24.3(3)
C37	4396.1(13)	3227.1(6)	1714.9(18)	29.7(4)
C38	4717.6(15)	2885.6(6)	1387(2)	37.1(5)
C39	4363.8(16)	2684.6(6)	523(2)	39.8(5)
C40	3686.6(15)	2838.8(6)	-8.5(18)	34.9(5)
C41	3368.8(13)	3183.3(6)	317.7(17)	29.2(4)
C42	4088.6(11)	4079.1(5)	949.7(15)	21.7(3)
C43	4018.2(11)	4158.0(5)	-193.1(15)	23.6(3)
C44	4584.9(11)	4375.6(5)	-753.0(15)	23.7(3)
C45	5271.7(12)	4519.4(6)	-217.4(16)	26.4(4)
C46	5370.4(12)	4434.6(6)	904.8(17)	26.8(4)
C47	4788.0(11)	4222.3(5)	1479.2(16)	24.5(3)
C48	5455(2)	2731.2(9)	1969(3)	60.8(8)
F1A	5741(3)	2421.1(11)	1583(4)	93.0(15)
F2A	5289(4)	2656(2)	3046(3)	136(3)
F3A	6054(3)	2981.1(12)	2125(5)	105.1(19)
F4A	5777(8)	2924(4)	2663(15)	105.1(19)
F5A	6041(6)	2646(3)	1151(9)	93.0(15)

Table 2 Fractional Atomic Coordinates (×10[4]) and Equivalent Isotropic Displacement Parameters (Å[2]×10[3]) for 2A-HFIPA_XW626. U_{eq} is defined as 1/3 of the trace of the orthogonalised U_{IJ} tensor.

Atom	*x*	*y*	*z*	U(eq)
F6A	5328(9)	2431(3)	2442(16)	120(5)
C49	3298.2(18)	2633.3(7)	-956(2)	47.9(6)
F1C	2574(3)	2826.2(16)	-1429(5)	67(2)
F2C	3055(6)	2316.6(15)	-845(4)	85(3)
F3C	3726(5)	2658(3)	-1921(6)	86(3)
F4C	3671(4)	2276.1(11)	-1033(4)	76.3(16)
F5C	3402(6)	2776.8(16)	-1852(4)	102(3)
F6C	2552(3)	2540.8(18)	-688(4)	90(2)
C50	3381.1(10)	3867.6(5)	2902.9(15)	20.9(3)
C51	3277.2(10)	3576.3(5)	3660.0(15)	22.7(3)
C52	3182.6(10)	3640.6(5)	4801.3(14)	23.2(3)
C53	3186.4(11)	3998.6(5)	5232.6(14)	23.3(3)
C54	3280.2(10)	4292.1(5)	4490.3(16)	23.0(3)
C55	3377.1(11)	4229.9(5)	3351.1(15)	22.4(3)
C56	2476.3(11)	3907.4(5)	1126.5(15)	23.0(3)
C57	2253.5(12)	4262.1(6)	743.5(17)	27.7(4)
C58	1453.5(12)	4346.0(6)	422(2)	32.9(4)
C59	831.8(12)	4081.6(6)	482(2)	32.9(4)
C60	1030.0(12)	3733.7(6)	883.7(18)	30.7(4)
C61	1834.6(12)	3648.1(5)	1200.6(15)	26.9(3)
C62	4419.3(13)	4477.1(6)	-1941.7(16)	28.8(4)
C63	6101.1(15)	4589.2(8)	1514(2)	42.3(5)
F1B	6374(9)	4379(4)	2341(14)	42(3)
F2B	6042(7)	4925(2)	1806(19)	74(4)
F3B	6776(4)	4581(4)	766(6)	55(2)
F4B	6629(9)	4765(6)	940(9)	99(5)
F5B	6449(8)	4341(4)	2165(12)	48(2)
F6B	5818(7)	4853(2)	2323(10)	71(2)
C64	3045.8(12)	3318.1(5)	5570.4(18)	30.3(4)
C65	3284.4(13)	4682.8(6)	4940.0(17)	29.2(4)
C66	1257.2(18)	4725.7(9)	-15(3)	56.1(8)
F1D	1847(3)	4990.6(12)	219(7)	81(2)
F2D	620(3)	4893.3(13)	753(4)	55.2(13)
F3D	947(6)	4771(2)	-912(5)	77(3)
F4D	587(3)	4706.2(16)	-739(5)	54.8(12)
F5D	1854(2)	4808.7(12)	-858(5)	81.8(19)
F6D	1210(5)	4972.2(10)	616(4)	91(3)
C67	380.3(15)	3438.2(8)	975(3)	44.0(6)

Table 2 Fractional Atomic Coordinates (×10^4) and Equivalent Isotropic Displacement Parameters (Å^2×10^3) for 2A-HFIPA_XW626. U_{eq} is defined as 1/3 of the trace of the orthogonalised U_{IJ} tensor.

Atom	*x*	*y*	*z*	U(eq)
B1	3413.3(12)	3808.1(5)	1544.6(17)	21.4(3)

Table 3 Anisotropic Displacement Parameters (Å^2×10^3) for 2A-HFIPA_XW626. The Anisotropic displacement factor exponent takes the form: $-2\pi^2[h^2a^{*2}U_{11}+2hka^*b^*U_{12}+\ldots]$.

Atom	U_{11}	U_{22}	U_{33}	U_{23}	U_{13}	U_{12}
Pd1	18.93(5)	18.73(5)	27.84(6)	-6.04(5)	3.41(5)	-0.17(4)
F1	52.8(10)	47.4(9)	133.9(19)	-29.7(12)	15.5(13)	-24.6(8)
F2	46.4(9)	67.7(12)	76.4(13)	21.3(10)	-3.4(9)	4.4(8)
F3	36.8(8)	74.8(13)	114.2(18)	-4.1(13)	-10.2(11)	1.0(8)
F4	51.5(11)	71.1(13)	122(2)	-5.4(13)	41.8(12)	-16.8(9)
F5	91.2(15)	70.2(13)	58.0(11)	8.8(10)	22.6(11)	9.3(11)
F6	79.4(12)	29.8(7)	88.4(13)	17.1(8)	24.2(12)	11.0(8)
F9	41.3(7)	74.7(11)	23.7(6)	0.6(6)	7.7(5)	-7.0(7)
F15	36.4(7)	48.1(8)	65.6(10)	19.5(8)	-3.5(7)	-20.3(6)
F16	87.7(12)	24.5(6)	39.4(7)	-6.3(5)	-20.8(8)	14.0(7)
F18	39.4(9)	86.3(15)	168(3)	36.8(17)	-45.8(13)	-25.8(9)
F19	56.5(9)	43.6(8)	29.6(7)	4.1(6)	-5.7(6)	18.0(7)
F20	55.6(9)	44.5(8)	24.4(6)	-6.0(5)	-10.7(6)	-4.1(7)
F23	101.3(15)	61.9(10)	32.0(7)	18.8(7)	-17.9(9)	-39.3(11)
F24	84.6(13)	51.9(10)	88.3(14)	43.2(10)	45.9(12)	39.2(9)
F25	67.3(11)	47.9(9)	68.7(12)	-29.6(8)	30.6(9)	-1.8(8)
F26	49.4(8)	29.9(6)	58.0(9)	-13.4(7)	-23.7(8)	-0.6(5)
F29	72.9(13)	92.5(16)	72.7(14)	10.2(12)	15.1(11)	-46.2(12)
F30	63.0(11)	65.1(12)	144(2)	-48.1(15)	27.4(15)	-32.9(10)
O1	28.1(6)	20.0(6)	37.7(7)	-7.0(6)	4.9(6)	-3.3(5)
O2	33.1(7)	20.3(6)	52.2(9)	-8.5(6)	5.9(7)	-4.0(6)
N1	18.9(6)	23.1(7)	30.5(8)	-5.1(6)	3.9(6)	-1.9(5)
N2	19.0(6)	21.0(7)	22.5(7)	-3.5(5)	0.9(5)	0.3(5)
C1	19.4(7)	27.0(9)	33.0(9)	-7.8(7)	5.7(7)	-3.3(6)
C2	24.0(9)	36.1(11)	38.4(11)	-0.9(8)	4.8(8)	-3.9(7)
C3	29.3(10)	53.0(14)	37.7(12)	-0.2(10)	9.7(9)	-7.1(9)
C4	23.0(9)	61.6(16)	43.4(13)	-9.4(11)	11.5(9)	-3.3(9)
C5	21.9(9)	60.4(16)	44.6(13)	-9.0(11)	2.7(9)	4.5(9)
C6	24.4(8)	40.1(10)	35.7(11)	-8.4(9)	2.9(8)	2.9(7)
C7	20.9(7)	21.1(8)	26.7(8)	-4.1(6)	2.2(6)	0.6(6)
C8	20.6(7)	21.8(8)	30.9(9)	-4.3(7)	2.3(7)	-1.8(6)

Table 3 Anisotropic Displacement Parameters (Å^2×10^3) for 2A-HFIPA_XW626. The Anisotropic displacement factor exponent takes the form: $-2\pi^2[h^2a^{*2}U_{11}+2hka^*b^*U_{12}+\ldots]$.

Atom	U_{11}	U_{22}	U_{33}	U_{23}	U_{13}	U_{12}
C9	29.9(9)	26.7(9)	44.8(12)	-12.7(8)	6.9(8)	-8.8(7)
C10	27.0(10)	63.8(16)	42.3(13)	12.2(11)	4.0(9)	1.1(10)
C11	48.3(15)	62.4(18)	57.8(17)	4.2(14)	-16.4(13)	0.8(13)
C12	30.5(10)	58.5(15)	36.4(12)	-4.2(10)	1.1(9)	11.4(10)
C13	58.6(18)	83(2)	42.2(15)	-12.7(15)	-11.9(13)	-2.6(16)
C14	31.9(10)	32.0(10)	42.8(12)	-14.8(8)	14.0(8)	-2.5(8)
C15	47.4(12)	33.9(10)	35.7(10)	-11.8(8)	9.3(10)	-3.9(10)
C16	41.6(11)	28.1(10)	42.0(12)	-14.0(8)	6.2(9)	-4.1(8)
C17	26.8(9)	18.5(8)	39.0(10)	-4.2(7)	-0.2(7)	0.7(6)
C18	20.5(8)	19.3(7)	25.7(8)	-2.0(6)	3.3(6)	0.6(6)
C19	32.2(9)	26.6(8)	26.2(8)	1.3(7)	2.6(6)	0.3(7)
C20	41.1(11)	36.2(11)	34.6(11)	8.4(9)	10.8(9)	-2.8(9)
C21	28.8(10)	35.0(11)	52.6(14)	7.2(10)	13.2(9)	-6.2(8)
C22	21.3(8)	30.0(10)	46.1(11)	1.4(8)	2.7(7)	-1.7(7)
C23	19.6(7)	19.9(7)	30.2(9)	-1.4(6)	2.0(6)	1.6(6)
C24	32.2(9)	24.6(9)	34.7(10)	-8.8(7)	10.7(8)	-0.6(7)
C25	68(2)	73(2)	50.8(17)	2.4(15)	4.7(15)	34.5(17)
C26	56.6(17)	60.5(18)	51.5(16)	8.0(14)	-8.8(14)	0.2(14)
C27	31.9(10)	22.7(9)	57.8(14)	-3.3(9)	11.1(10)	-2.0(7)
C28	35.2(12)	38.3(13)	96(2)	-8.0(14)	14.3(14)	-7.3(10)
C29	44.4(11)	41.8(11)	28.9(10)	3.7(9)	-6.1(8)	-2.4(10)
C30	46.8(15)	64.9(17)	45.0(14)	1.6(12)	-16.0(11)	14.4(13)
C31	23.4(8)	27.4(9)	24.8(8)	-1.6(6)	-0.5(6)	2.2(6)
C32	74.4(18)	29.7(11)	32.0(11)	6.3(8)	7.6(11)	13.3(11)
C33	53.3(14)	34.6(11)	59.8(16)	8.1(10)	13.6(14)	3.4(11)
C34	77(2)	72(2)	29.4(12)	1.4(13)	-5.0(12)	1.8(18)
C35	46.7(12)	45.3(13)	37.6(12)	-2.2(10)	-14.5(10)	-7.0(10)
C36	27.5(8)	22.8(8)	22.6(8)	-4.5(6)	-0.5(6)	-3.2(6)
C37	33.7(9)	25.0(9)	30.4(9)	-7.9(7)	-4.0(8)	3.9(7)
C38	42.3(11)	28.2(10)	40.9(12)	-6.4(8)	-0.1(9)	8.7(8)
C39	52.7(12)	25.5(9)	41.3(11)	-10.3(9)	8.8(11)	0.7(8)
C40	45.7(12)	28.7(10)	30.2(10)	-13.2(8)	7.8(9)	-11.3(9)
C41	33.6(9)	28.0(9)	26.1(9)	-7.5(7)	-0.8(7)	-5.7(7)
C42	21.6(7)	20.3(8)	23.2(8)	-3.1(6)	-2.7(6)	1.2(6)
C43	23.4(8)	26.4(8)	21.2(8)	-5.9(6)	-3.1(6)	1.6(6)
C44	25.4(8)	26.4(8)	19.5(8)	-2.8(6)	-0.2(6)	4.3(6)
C45	23.3(8)	29.2(9)	26.6(9)	1.1(7)	-0.3(6)	-0.4(7)
C46	22.7(8)	29.4(9)	28.3(9)	1.2(7)	-5.5(7)	-2.3(7)
C47	25.3(8)	26.1(8)	22.0(8)	0.4(6)	-5.7(6)	-0.4(6)

Table 3 Anisotropic Displacement Parameters ($Å^2×10^3$) for 2A-HFIPA_XW626. The Anisotropic displacement factor exponent takes the form: $-2\pi^2[h^2a^{*2}U_{11}+2hka^*b^*U_{12}+…]$.

Atom	U_{11}	U_{22}	U_{33}	U_{23}	U_{13}	U_{12}
C48	64.1(18)	47.7(15)	71(2)	-14.0(14)	-13.6(15)	29.8(14)
F1A	96(3)	65(2)	117(3)	-34(2)	-28(2)	57(2)
F2A	131(4)	208(6)	68(2)	27(3)	-7(2)	109(5)
F3A	61(3)	68(2)	187(5)	10(3)	-59(3)	6.8(18)
F4A	61(3)	68(2)	187(5)	10(3)	-59(3)	6.8(18)
F5A	96(3)	65(2)	117(3)	-34(2)	-28(2)	57(2)
F6A	134(11)	48(5)	177(14)	41(7)	-63(9)	15(6)
C49	59.2(16)	40.4(12)	44.0(13)	-23.6(10)	5.5(11)	-13.9(11)
F1C	61(3)	72(3)	69(3)	-44(3)	-30(2)	8(2)
F2C	162(8)	40(3)	54(3)	5(2)	-34(4)	-57(4)
F3C	73(4)	136(8)	48(3)	-50(4)	22(3)	-46(4)
F4C	115(4)	43.2(19)	70(2)	-36.3(17)	-32(3)	14(2)
F5C	223(10)	52(3)	30(2)	10.5(19)	-47(4)	-50(4)
F6C	61(2)	114(5)	94(4)	-67(4)	-5(2)	-31(2)
C50	19.6(7)	20.3(7)	22.7(8)	-3.2(6)	-3.3(6)	-0.8(6)
C51	21.1(7)	19.8(7)	27.4(8)	-0.4(6)	-2.7(6)	-2.5(6)
C52	19.0(6)	25.6(7)	25.1(7)	2.5(6)	-2.7(6)	-2.9(7)
C53	19.3(7)	30.3(8)	20.4(7)	-2.8(6)	-2.6(6)	-0.2(7)
C54	22.2(7)	23.0(7)	23.8(7)	-5.8(7)	-3.5(6)	0.0(5)
C55	23.8(7)	21.2(8)	22.3(8)	-2.0(6)	-3.1(6)	1.0(6)
C56	23.8(8)	26.2(8)	18.8(8)	-2.8(6)	-2.4(6)	-0.8(6)
C57	25.4(8)	27.5(9)	30.1(10)	0.2(7)	-0.9(7)	0.0(7)
C58	27.8(8)	35.4(10)	35.3(10)	7.1(9)	-0.2(8)	4.7(7)
C59	22.7(8)	46.5(11)	29.4(9)	1.5(9)	-3.3(8)	1.9(7)
C60	23.9(8)	40.4(11)	27.9(9)	-1.2(8)	-3.3(7)	-5.8(7)
C61	25.5(7)	29.4(8)	25.9(8)	1.1(6)	-3.6(7)	-4.2(8)
C62	31.7(9)	33.8(10)	20.9(8)	-2.5(7)	0.1(7)	2.1(8)
C63	34.1(11)	50.2(13)	42.6(12)	10.3(10)	-14.9(9)	-13.9(10)
F1B	36(4)	65(5)	26(3)	11(4)	-10(2)	-18(4)
F2B	60(4)	37(3)	126(10)	-24(4)	-48(5)	-4(2)
F3B	24(2)	99(5)	42(2)	13(3)	-7.2(14)	-24(3)
F4B	83(5)	154(11)	58(4)	45(5)	-30(4)	-91(7)
F5B	31(2)	72(3)	40(5)	0(3)	-17(3)	5(2)
F6B	73(4)	51(3)	90(5)	-21(3)	-44(3)	-11(3)
C64	26.9(8)	31.8(9)	32.2(9)	7.7(8)	-1.3(8)	-3.5(7)
C65	33.2(10)	26.9(9)	27.6(9)	-7.5(7)	-4.5(7)	3.3(7)
C66	38.0(13)	45.2(15)	85(2)	22.3(14)	-10.9(13)	7.5(11)
F1D	38(2)	39(2)	166(7)	37(3)	-12(3)	1.6(17)
F2D	59(3)	49(2)	58(3)	2.9(17)	8(2)	20(2)

Table 3 Anisotropic Displacement Parameters (Å^2×10^3) for 2A-HFIPA_XW626. The Anisotropic displacement factor exponent takes the form: $-2\pi^2[h^2a^{*2}U_{11}+2hka^*b^*U_{12}+\ldots]$.

Atom	U_{11}	U_{22}	U_{33}	U_{23}	U_{13}	U_{12}
F3D	145(8)	50(3)	35(2)	11(2)	-6(4)	25(5)
F4D	53(2)	53(2)	58(3)	12.3(18)	-12.6(18)	21.7(18)
F5D	59(2)	62(2)	125(4)	63(3)	19(2)	17.4(19)
F6D	147(7)	35.3(18)	92(3)	-20(2)	-47(4)	31(3)
C67	27.2(10)	48.7(14)	55.9(15)	1.8(11)	-5.3(10)	-10.4(9)
B1	22.5(8)	20.9(8)	20.8(8)	-2.2(7)	-2.9(7)	-1.0(6)

Table 4 Bond Lengths for 2A-HFIPA_XW626.

Atom	Atom	Length/Å	Atom	Atom	Length/Å
Pd1	O1	2.0577(13)	C36	C37	1.398(3)
Pd1	N1	2.0208(15)	C36	C41	1.401(3)
Pd1	N2	2.1292(15)	C36	B1	1.643(3)
Pd1	C14	2.024(2)	C37	C38	1.391(3)
F1	C28	1.332(3)	C38	C39	1.388(3)
F2	C33	1.329(4)	C38	C48	1.494(4)
F3	C28	1.322(4)	C39	C40	1.388(4)
F4	C28	1.325(4)	C40	C41	1.398(3)
F5	C33	1.323(4)	C40	C49	1.496(3)
F6	C33	1.332(3)	C42	C43	1.404(3)
F9	C62	1.334(2)	C42	C47	1.401(2)
F15	C64	1.326(2)	C42	B1	1.632(3)
F16	C65	1.326(2)	C43	C44	1.383(3)
F18	C67	1.326(3)	C44	C45	1.388(3)
F19	C62	1.351(2)	C44	C62	1.496(3)
F20	C62	1.336(2)	C45	C46	1.389(3)
F23	C64	1.333(3)	C46	C47	1.398(3)
F24	C64	1.320(3)	C46	C63	1.502(3)
F25	C65	1.345(3)	C48	F1A	1.294(4)
F26	C65	1.330(2)	C48	F2A	1.346(6)
F29	C67	1.325(4)	C48	F3A	1.338(5)
F30	C67	1.314(4)	C48	F4A	1.203(13)
O1	C17	1.219(2)	C48	F5A	1.402(10)
O2	C17	1.343(2)	C48	F6A	1.236(10)
O2	C27	1.429(3)	C49	F1C	1.480(5)
N1	C1	1.442(2)	C49	F2C	1.213(5)
N1	C8	1.296(2)	C49	F3C	1.352(7)
N2	C7	1.287(2)	C49	F4C	1.423(5)

Table 4 Bond Lengths for 2A-HFIPA_XW626.

Atom	Atom	Length/Å	Atom	Atom	Length/Å
N2	C18	1.434(2)	C49	F5C	1.204(5)
C1	C2	1.402(3)	C49	F6C	1.299(5)
C1	C6	1.402(3)	C50	C51	1.396(2)
C2	C3	1.397(3)	C50	C55	1.409(2)
C2	C10	1.520(3)	C50	B1	1.643(3)
C3	C4	1.375(4)	C51	C52	1.396(2)
C4	C5	1.383(4)	C52	C53	1.387(2)
C5	C6	1.390(3)	C52	C64	1.498(3)
C6	C12	1.522(4)	C53	C54	1.389(3)
C7	C8	1.505(2)	C54	C55	1.393(3)
C7	C24	1.493(3)	C54	C65	1.505(2)
C8	C9	1.497(3)	C56	C57	1.403(3)
C10	C11	1.526(4)	C56	C61	1.402(2)
C10	C25	1.535(5)	C56	B1	1.643(3)
C12	C13	1.536(4)	C57	C58	1.390(3)
C12	C26	1.513(5)	C58	C59	1.389(3)
C14	C15	1.517(3)	C58	C66	1.497(3)
C15	C16	1.521(3)	C59	C60	1.379(3)
C16	C17	1.491(3)	C60	C61	1.397(3)
C18	C19	1.407(3)	C60	C67	1.502(3)
C18	C23	1.401(3)	C63	F1B	1.323(11)
C19	C20	1.398(3)	C63	F2B	1.262(7)
C19	C29	1.524(3)	C63	F3B	1.417(6)
C20	C21	1.385(4)	C63	F4B	1.269(7)
C21	C22	1.377(3)	C63	F5B	1.314(9)
C22	C23	1.397(3)	C63	F6B	1.432(8)
C23	C31	1.522(3)	C66	F1D	1.380(6)
C27	C28	1.510(4)	C66	F2D	1.511(5)
C27	C33	1.524(4)	C66	F3D	1.199(7)
C29	C30	1.531(4)	C66	F4D	1.396(6)
C29	C34	1.529(3)	C66	F5D	1.433(5)
C31	C32	1.525(3)	C66	F6D	1.168(5)
C31	C35	1.521(3)			

Table 5 Bond Angles for 2A-HFIPA_XW626.

Atom	Atom	Atom	Angle/°	Atom	Atom	Atom	Angle/°
O1	Pd1	N2	93.95(6)	F1A	C48	F2A	104.1(4)
N1	Pd1	O1	171.30(6)	F1A	C48	F3A	111.5(4)

Table 5 Bond Angles for 2A-HFIPA_XW626.

Atom	Atom	Atom	Angle/°	Atom	Atom	Atom	Angle/°
N1	Pd1	N2	77.99(6)	F2A	C48	C38	111.2(3)
N1	Pd1	C14	99.00(8)	F3A	C48	C38	113.6(3)
C14	Pd1	O1	89.27(7)	F3A	C48	F2A	98.4(5)
C14	Pd1	N2	174.96(9)	F4A	C48	C38	117.2(7)
C17	O1	Pd1	130.36(14)	F4A	C48	F5A	108.3(9)
C17	O2	C27	117.15(16)	F4A	C48	F6A	105.0(10)
C1	N1	Pd1	121.66(12)	F5A	C48	C38	107.5(5)
C8	N1	Pd1	116.69(12)	F6A	C48	C38	113.8(6)
C8	N1	C1	121.55(15)	F6A	C48	F5A	104.2(9)
C7	N2	Pd1	114.37(12)	F1C	C49	C40	113.3(2)
C7	N2	C18	124.11(15)	F2C	C49	C40	121.2(3)
C18	N2	Pd1	121.15(11)	F2C	C49	F1C	102.8(5)
C2	C1	N1	117.95(18)	F2C	C49	F3C	108.8(5)
C6	C1	N1	118.96(19)	F3C	C49	C40	113.6(4)
C6	C1	C2	122.87(19)	F3C	C49	F1C	92.9(5)
C1	C2	C10	122.61(19)	F4C	C49	C40	108.4(3)
C3	C2	C1	117.1(2)	F5C	C49	C40	114.0(3)
C3	C2	C10	120.3(2)	F5C	C49	F4C	105.6(4)
C4	C3	C2	121.5(2)	F5C	C49	F6C	117.5(5)
C3	C4	C5	119.8(2)	F6C	C49	C40	109.4(3)
C4	C5	C6	121.9(2)	F6C	C49	F4C	100.5(4)
C1	C6	C12	122.48(19)	C51	C50	C55	116.42(16)
C5	C6	C1	116.9(2)	C51	C50	B1	123.39(15)
C5	C6	C12	120.6(2)	C55	C50	B1	119.90(16)
N2	C7	C8	114.39(16)	C50	C51	C52	121.75(17)
N2	C7	C24	125.49(17)	C51	C52	C64	119.42(17)
C24	C7	C8	120.11(16)	C53	C52	C51	121.24(16)
N1	C8	C7	116.28(16)	C53	C52	C64	119.31(16)
N1	C8	C9	124.27(17)	C52	C53	C54	117.81(15)
C9	C8	C7	119.45(16)	C53	C54	C55	121.25(16)
C2	C10	C11	112.8(2)	C53	C54	C65	118.69(17)
C2	C10	C25	109.8(2)	C55	C54	C65	120.06(17)
C11	C10	C25	110.3(2)	C54	C55	C50	121.53(17)
C6	C12	C13	112.1(2)	C57	C56	B1	122.46(16)
C26	C12	C6	111.6(2)	C61	C56	C57	115.65(17)
C26	C12	C13	109.6(3)	C61	C56	B1	121.76(16)
C15	C14	Pd1	110.16(15)	C58	C57	C56	121.98(18)
C14	C15	C16	114.3(2)	C57	C58	C66	119.6(2)
C17	C16	C15	113.67(17)	C59	C58	C57	121.22(19)
O1	C17	O2	119.48(19)	C59	C58	C66	119.1(2)

Table 5 Bond Angles for 2A-HFIPA_XW626.

Atom	Atom	Atom	Angle/°	Atom	Atom	Atom	Angle/°
O1	C17	C16	126.68(19)	C60	C59	C58	117.95(18)
O2	C17	C16	113.84(17)	C59	C60	C61	120.92(18)
C19	C18	N2	119.65(16)	C59	C60	C67	120.19(19)
C23	C18	N2	117.08(16)	C61	C60	C67	118.9(2)
C23	C18	C19	122.87(16)	C60	C61	C56	122.25(18)
C18	C19	C29	121.18(17)	F9	C62	F19	106.60(18)
C20	C19	C18	116.90(19)	F9	C62	F20	107.06(17)
C20	C19	C29	121.76(19)	F9	C62	C44	113.28(17)
C21	C20	C19	121.2(2)	F19	C62	C44	111.06(16)
C22	C21	C20	120.6(2)	F20	C62	F19	105.77(17)
C21	C22	C23	121.1(2)	F20	C62	C44	112.60(17)
C18	C23	C31	121.18(16)	F1B	C63	C46	114.8(7)
C22	C23	C18	117.36(18)	F1B	C63	F3B	101.7(8)
C22	C23	C31	121.46(18)	F2B	C63	C46	115.4(4)
O2	C27	C28	105.8(2)	F2B	C63	F1B	111.4(10)
O2	C27	C33	108.78(19)	F2B	C63	F3B	104.7(6)
C28	C27	C33	113.7(2)	F3B	C63	C46	107.3(4)
F1	C28	C27	112.6(2)	F4B	C63	C46	117.1(4)
F3	C28	F1	107.7(3)	F4B	C63	F5B	111.7(9)
F3	C28	F4	107.6(3)	F4B	C63	F6B	104.8(6)
F3	C28	C27	109.7(2)	F5B	C63	C46	112.2(6)
F4	C28	F1	108.1(3)	F5B	C63	F6B	100.7(7)
F4	C28	C27	111.0(3)	F6B	C63	C46	108.6(4)
C19	C29	C30	110.1(2)	F15	C64	F23	105.6(2)
C19	C29	C34	113.5(2)	F15	C64	C52	112.56(16)
C34	C29	C30	109.6(2)	F23	C64	C52	112.73(17)
C23	C31	C32	111.04(16)	F24	C64	F15	106.48(19)
C35	C31	C23	113.76(17)	F24	C64	F23	106.4(2)
C35	C31	C32	109.95(19)	F24	C64	C52	112.50(18)
F2	C33	F6	106.9(2)	F16	C65	F25	106.08(18)
F2	C33	C27	110.2(2)	F16	C65	F26	107.59(19)
F5	C33	F2	108.1(3)	F16	C65	C54	113.32(16)
F5	C33	F6	108.2(2)	F25	C65	C54	111.63(18)
F5	C33	C27	111.0(2)	F26	C65	F25	104.88(17)
F6	C33	C27	112.3(3)	F26	C65	C54	112.76(16)
C37	C36	C41	115.83(18)	C58	C66	F2D	107.3(3)
C37	C36	B1	120.13(16)	F1D	C66	C58	114.2(3)
C41	C36	B1	123.64(17)	F1D	C66	F2D	94.4(4)
C38	C37	C36	122.51(19)	F3D	C66	C58	121.8(5)
C37	C38	C48	119.8(2)	F3D	C66	F1D	112.4(5)

Table 5 Bond Angles for 2A-HFIPA_XW626.

Atom	Atom	Atom	Angle/°	Atom	Atom	Atom	Angle/°
C39	C38	C37	121.0(2)	F3D	C66	F2D	101.8(5)
C39	C38	C48	119.2(2)	F4D	C66	C58	109.8(3)
C38	C39	C40	117.6(2)	F4D	C66	F5D	95.7(4)
C39	C40	C41	121.3(2)	F5D	C66	C58	107.0(3)
C39	C40	C49	119.1(2)	F6D	C66	C58	118.7(4)
C41	C40	C49	119.6(2)	F6D	C66	F4D	113.0(4)
C40	C41	C36	121.8(2)	F6D	C66	F5D	110.1(5)
C43	C42	B1	119.48(16)	F18	C67	C60	112.6(2)
C47	C42	C43	115.75(18)	F29	C67	F18	105.6(3)
C47	C42	B1	124.59(16)	F29	C67	C60	112.4(2)
C44	C43	C42	122.26(17)	F30	C67	F18	108.1(3)
C43	C44	C45	121.49(17)	F30	C67	F29	104.8(3)
C43	C44	C62	118.73(17)	F30	C67	C60	112.8(2)
C45	C44	C62	119.66(18)	C36	B1	C56	113.32(14)
C44	C45	C46	117.35(18)	C42	B1	C36	104.16(14)
C45	C46	C47	121.25(18)	C42	B1	C50	112.13(14)
C45	C46	C63	118.79(19)	C42	B1	C56	111.16(15)
C47	C46	C63	119.90(19)	C50	B1	C36	112.16(15)
C46	C47	C42	121.84(18)	C50	B1	C56	104.15(14)
F1A	C48	C38	116.2(3)				

Table 6 Hydrogen Atom Coordinates (Å×10[4]) and Isotropic Displacement Parameters (Å[2]×10[3]) for 2A-HFIPA_XW626.

Atom	*x*	*y*	*z*	U(eq)
H3	10283.97	4373.94	2649.45	48
H4	11504.95	4175.44	3447.16	51
H5	11523.84	3963.99	5276.11	51
H9A	9470.35	4703.55	6191.86	51
H9B	9107.58	4689.63	7431.93	51
H9C	8604.45	4894.67	6463.47	51
H10	8276.77	4299.02	3896.86	53
H11A	8092.75	4297.37	1969.9	84
H11B	8685.32	3957.98	2286.01	84
H11C	9062.35	4330.61	1764.81	84
H12	9864.41	3990.72	7202.62	50
H13A	11594.42	3850.3	7049.48	92
H13B	11089	3917.63	8176.83	92
H13C	11220.54	4252.41	7315.37	92

Table 6 Hydrogen Atom Coordinates (Å×10⁴) and Isotropic Displacement Parameters (Å²×10³) for 2A-HFIPA_XW626.

Atom	*x*	*y*	*z*	U(eq)
H14A	9101.7	3563.16	3844.2	43
H14B	9150.72	3183.97	4544.96	43
H15A	8489.23	3128.81	2729.3	47
H15B	7748.04	3369.95	3221.92	47
H16A	7395.16	2741.65	3284.53	45
H16B	8210.42	2645.45	3971.55	45
H20	5890.02	3370.28	9341.41	45
H21	4771.76	3326.76	8144.02	47
H22	4857.42	3545.85	6332.32	39
H24A	7269.52	4751.55	7278.57	46
H24B	7682.55	4558.98	8343.63	46
H24C	6797.65	4424.8	7920.44	46
H25A	9179.39	4911.69	2977.2	96
H25B	8719.03	4913.58	4156.9	96
H25C	8196.76	4894.27	3027.1	96
H26A	9701.01	3391.16	6467.29	84
H26B	10219.54	3374.78	7600.67	84
H26C	10677.63	3338.41	6424.37	84
H27	6348.66	2896	6459.81	45
H29	7728.14	3869.54	8874.3	46
H30A	8182.76	3347.92	7876.21	78
H30B	7733.06	3074.41	8734.69	78
H30C	8503.43	3317.14	9134.76	78
H31	6691.63	3870.71	5008.23	30
H32A	5941.52	4384.61	4416.61	68
H32B	6147.66	4433.78	5713.42	68
H32C	5246.56	4307.64	5333.02	68
H34A	6834.81	3787.6	10445.82	90
H34B	7743.29	3634.3	10673.66	90
H34C	7013.48	3350.13	10398.53	90
H35A	5773.49	3729.75	3627.21	65
H35B	5028.66	3693.66	4492.8	65
H35C	5745.8	3388.85	4484.72	65
H37	4653.21	3357.06	2310.08	36
H39	4577.46	2449.78	303.82	48
H41	2909.45	3282.58	-71	35
H43	3565.68	4058.44	-595.55	28
H45	5658.88	4669.72	-603.12	32
H47	4868.92	4173.67	2250.33	29

Table 6 Hydrogen Atom Coordinates (Å×10⁴) and Isotropic Displacement Parameters (Å²×10³) for 2A-HFIPA_XW626.

Atom	*x*	*y*	*z*	U(eq)
H51	3270.66	3327.68	3391.43	27
H53	3126.72	4041.62	6010.18	28
H55	3442.01	4436.49	2865.08	27
H57	2661.69	4450.68	702.78	33
H59	287.03	4138.92	253.39	39
H61	1951.04	3405.97	1475.76	32

Table 7 Atomic Occupancy for 2A-HFIPA_XW626.

Atom	*Occupancy*	Atom	*Occupancy*	Atom	*Occupancy*
F1A	0.727(5)	F2A	0.727(5)	F3A	0.727(5)
F4A	0.273(5)	F5A	0.273(5)	F6A	0.273(5)
F1C	0.437(8)	F2C	0.437(8)	F3C	0.437(8)
F4C	0.563(8)	F5C	0.563(8)	F6C	0.563(8)
F1B	0.44(3)	F2B	0.44(3)	F3B	0.44(3)
F4B	0.56(3)	F5B	0.56(3)	F6B	0.56(3)
F1D	0.443(7)	F2D	0.443(7)	F3D	0.443(7)
F4D	0.557(7)	F5D	0.557(7)	F6D	0.557(7)
